# A tumor-specific endogenous repetitive element is induced by herpesviruses

**DOI:** 10.1038/s41467-018-07944-x

**Published:** 2019-01-09

**Authors:** Maciej T. Nogalski, Alexander Solovyov, Anupriya S. Kulkarni, Niyati Desai, Adam Oberstein, Arnold J. Levine, David T. Ting, Thomas Shenk, Benjamin D. Greenbaum

**Affiliations:** 1 Department of Molecular Biology, Princeton University, Princeton, NJ 08544 USA; 2 Department of Medicine, Hematology and Medical Oncology, Tisch Cancer Institute, Icahn School of Medicine at Mount Sinai, New York, NY 10029 USA; 3 Department of Oncological Sciences, Tisch Cancer Institute, Icahn School of Medicine at Mount Sinai, New York, NY 10029 USA; 4 Department of Pathology, Tisch Cancer Institute, Icahn School of Medicine at Mount Sinai, New York, NY 10029 USA; 5 Massachusetts General Hospital Cancer Center, Harvard Medical School, Charlestown, MA 02129 USA; 6 The Simons Center for Systems Biology, School of Natural Sciences, Institute for Advanced Study, Princeton, NJ 08540 USA; 7 Precision Immunology Institute, Icahn School of Medicine at Mount Sinai, New York, NY 10029 USA; 8 Icahn Institute for Data Science and Genomic Technology, Icahn School of Medicine at Mount Sinai, New York, NY 10029 USA

## Abstract

Tandem satellite repeats account for 3% of the human genome. One of them, Human Satellite II (HSATII), is highly expressed in several epithelial cancers and cancer cell lines. Here we report an acute induction of HSATII RNA in human cells infected with two herpes viruses. We show that human cytomegalovirus (HCMV) IE1 and IE2 proteins cooperate to induce HSATII RNA affecting several aspects of the HCMV replication cycle, viral titers and infected-cell processes. HSATII RNA expression in tissue from two chronic HCMV colitis patients correlates with the strength of CMV antigen staining. Thus, endogenous HSATII RNA synthesis after herpesvirus infections appears to have functionally important consequences for viral replication and may provide a novel insight into viral pathogenesis. The HSATII induction seen in both infected and cancer cells suggests possible convergence upon common HSATII-based regulatory mechanisms in these seemingly disparate diseases.

## Abstract (web summary)

The human genome includes a large amount of repetitive sequence, such as human satellite II (HSATII), but their function remains largely unknown. Here, Nogalski et al. show that herpesvirus infection induces HSATII RNA expression, which in turn affects virus replication and cell motility.

## Introduction

Repetitive sequences account for more than 50% of the human genome with tandem satellite repeats comprising approximately 3%^1^. Although repetitive sequences are ubiquitous, there is a limited understanding of their functions. Satellite DNA, satDNA, were shown to form centromeric and pericentromeric loci, and have been implicated in chromosome organization and segregation, kinetochore formation, and heterochromatin regulation^2^. Developments in next-generation sequencing (NSG) showed these genomic sites, previously thought to be largely transcriptionally inert, could produce RNA transcripts which contribute to the role of satDNA in chromosome and heterochromatin function^3^.

Human satellite repeat II (HSATII) and its mouse counterpart (GSAT) were further shown to be highly expressed in several epithelial cancers but not corresponding normal tissue^4,5^. While some satellite repeat transcription was found to be stress-dependent^6^ or triggered during cellular apoptosis, differentiation, or senescence^7,8^; HSATII transcription was refractory to these generalized environmental stressors and was induced when cancer cells were grown in non-adherent conditions or as xenografts in mice^9^. The sequence motifs of HSATII RNA mimic specifically some zoonotic viruses by containing CpG motifs within an AU-rich sequence context. These types of sequences are vastly under-represented in the human genome, avoided in viruses^10^, immune-stimulatory in cells^5,11^, and sensed by the antiviral protein ZAP if present in viral RNA^12^.

Human cytomegalovirus (HCMV), like all herpesviruses, causes a chronic infection with lifelong latency in humans. HCMV is a leading opportunistic pathogen in immunosuppressed individuals, with infection capable of causing birth defects^13^. HCMV strongly modulates cellular homeostasis for optimal viral replication and spread, and can be reactivated in the setting of reduced immunosurveillance^13^, an immunological feature also observed in the emergence of cancers^14^. We therefore sought to determine if HSATII expression plays a role in virus infections and contributes to viral fitness.

Our study shows herpesvirus infected cells have drastically induced HSATII RNA levels. In the case of HCMV, we report that accumulation of HSATII RNA requires the combined action of the viral IE1 and IE2 proteins and that HSATII RNA is important for efficient viral protein expression and localization, viral replication, and release of infectious particles. Moreover, our work depicts HSATII RNA as a regulator of several cellular processes, such as cellular motility, and provides a potential link between increased HSATII expression and virus-mediated pathobiology in CMV colitis.

## Results

### HSATII RNA accumulation is induced by herpesvirus infection

**Fig. 1:**
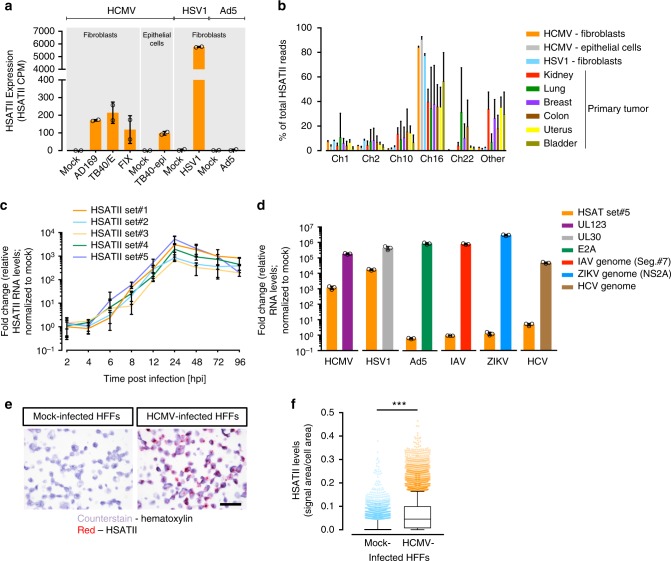
HCMV and HSV1, but not ADV, FLU, ZIKV, HCV, induce HSATII expression. HFFs were infected with HCMV (3 TCID_50_/cell), HSV (3 TCID_50_/cell), or Ad5 (10 FFU/cell), and RNA samples were collected at 48, 9, or 24 hpi, respectively. RNA was isolated and analyzed using RNA-seq. **a** HSATII expression in terms of counts per million reads (CPM) was computed and normalized across samples. *n* = 2. **b** HSATII chromosomal origin in infected cells or primary tumors was depicted based on the number of unique HSATII reads mapped to specific chromosomal loci. Data are presented as a percentage of total HSATII reads mean ± SD. *n* = 2. Open circles represent single data points. **c** HFFs were infected with HCMV (TB40/E-GFP) at 3 TCID_50_/cell and RNA samples were collected at the indicated times. HSATII-specific primers were used in RT-qPCR analysis. GAPDH was used as an internal control. Data are presented as a fold change mean ± SD. *n* = 3. **d** Fibroblasts were infected with HCMV (3 TCID_50_/cell), HSV1 (3 TCID_50_/cell), Ad5 (10 FFU/cell), FLU (3 TCID_50_/cell), or ZIKV (10 PFU/cell), and Huh7 cells were infected with HCV (1 TCID_50_/cell). RNA samples were collected at 9 hpi (HSV) or 24 hpi (all other viruses). HSATII-specific primers were used in RT-qPCR analysis. Viral infection was controlled by probing for a presence of viral transcripts: UL123 (HCMV), UL30 (HSV1), E2A (Ad5) or viral genomes: IAV and ZIKV. GAPDH was used as an internal control. Data are presented as a fold change mean ± SD. *n* = 3. Open circles represent single data points. **e** Mock- and HCMV (TB40/E-GFP)-infected HFFs at 3 TCID_50_/cell were collected at 24 hpi and HSATII RNA was visualized by ISH assay. Nuclei were counterstained with hematoxylin and HSATII is shown as red dots. Scale bar: 50 µm. **f** HSATII signal from ISH staining was quantified based on the ratio of HSATII signal area to cell area using BDZ 6.0 software and is presented in box plots (a central line shows median and bounds of box the 25th and 75th percentiles) with 10–90 percentile whiskers. Dots represent outliers. *n* = 3. ***P < 0.001 by the unpaired, two-tailed *t*-test

We performed total RNA-seq to capture both coding and non-coding transcriptomes of acute HCMV infection in human foreskin fibroblasts (HFFs) (Supplementary Fig. 1a). With a focus on non-coding RNAs whose levels changed with infection, we found the majority of transcripts (74%) were downregulated at 48 hpi, and this tendency was the most profound for repetitive elements as 87% of them were decreased in HCMV-infected cells. Of the 13% of repeat elements upregulated upon infection, there was a striking (≥100-fold) increase of HSATII RNA over that seen in mock-infected cells (Fig. 1a and Supplementary Fig. 1b). Importantly, the ability to induce HSATII expression was common for both the HCMV laboratory strain (AD169) and the more clinically relevant isolates (TB40/E and FIX) (Fig. 1a). As HSATII induction could be an indiscriminate cellular response to any infection, we tested HSATII expression in the same cell type infected with two other DNA viruses, herpes simplex virus (HSV1), an α-herpesvirus, and adenovirus (Ad5). HSV1 increased HSATII transcript levels to an even greater extent (>1500-fold) but, interestingly, Ad5 did not alter the expression of the satellite RNA (Fig. 1a). By analyzing only uniquely mapped HSATII reads in the RNA-seq dataset, our data suggest that HSATII in infected cells is produced preferentially from chromosome 1, 2, 10, and 16 and that HSATII accumulation from chromosome 16 was heavily favored following infection (Fig. 1b)—with the caveat that repeats often have high genomic diversity, abundant integration sites, and incomplete annotation. Of note, infected cells seem to have less diverse HSATII chromosomal expression patterns when compared to primary tumors.

HSATII sequences were found to be often expressed in some cancers from the chromosome 7 locus^15^. Our analysis determined that in tumors a higher percentage of HSATII transcripts also originated from chromosome 22 as well as other chromosomal loci (Fig. 1b). However, the preferential expression of HSATII in infected cells closely aligned with chromosomes where HSATII is a main constituent of the pericentromere^9^ and which are largely responsible for the HSATII expression observed in cancer cells (Fig. 1b).

To validate our RNA-seq analysis, we designed sets of HSATII-specific PCR primers (HSATII Set#1–#5) based on highly expressed transcripts detected in HCMV-infected cells. Analysis of the kinetics of HSATII transcript accumulation in HCMV-infected fibroblasts demonstrated an initial induction during the immediate-early phase of infection at 6 hpi with a continued increase up to the onset of viral DNA replication at 24 hpi (Fig. 1c). HSATII levels then decreased, but remained substantially elevated until the end of the viral replication cycle at 96 hpi. Interestingly, the kinetics of HSATII expression were cell type-specific. In HCMV-infected ARPE-19 epithelial cells, HSATII expression was accelerated and reached the maximum at 12 hpi (Supplementary Fig. 2). HSATII RNA was also induced in fibroblasts infected with HSV1; but Ad5, as well as several RNA viruses—influenza A (IAV), ZIKA virus (ZIKV), and hepatitis C virus (HCV)—failed to induce the probed HSATII sequences (Fig. 1d), even when close to 100% of cells were infected (Supplementary Fig. 3). Thus, our data suggest that HSATII induction might be specific to herpesvirus infections.

The detection of HSATII transcripts required a reverse transcription step before PCR amplification (Supplementary Fig. 4), suggesting that HSATII transcripts in HCMV-infected cells do not create RNA-derived DNA intermediates, as observed in cancer cells^9^. Perhaps the rapid HSATII induction or lack of reverse transcriptase activity in HCMV-infected cells, as opposed to malignant cells, may prevent the generation of DNA-containing intermediates. Moreover, in contrast to a control HCMV mRNA, UL123, HSATII RNA from infected cells was not retained on an oligo-dT matrix or efficiently amplified from oligo dT-based cDNA, indicating that it is predominantly not polyadenylated (Supplementary Fig. 5). The lack of a polyA tail on HSATII transcripts was confirmed by inspecting unique HSATII reads, and emphasizes the need for total RNA-sequencing to broadly explore this phenomena^16^. HSATII expression was also analyzed in mock- and HCMV-infected cells using RNA in situ hybridization (ISH) for detection of HSATII RNA^4^. HCMV-infected cells showed a robust increase in a signal for HSATII RNA with the majority of signal localized in nuclei (Fig. 1e, f).

### HCMV IE1 and IE2 proteins induce HSATII expression

**Fig. 2:**
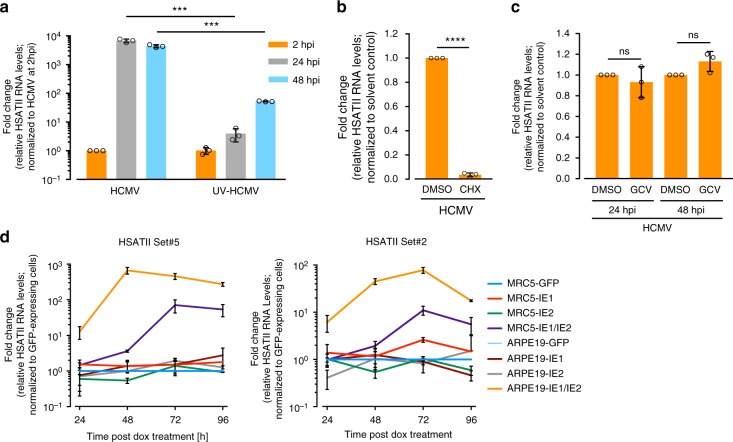
Accumulation of HSATII RNA in HCMV-infected cells requires both IE1 and IE2 viral proteins. **a** HFFs were infected with untreated or UV-irradiated HCMV (TB40/E-GFP) at 3 TCID_50_/cell, RNA samples were collected at specified times. **b** HFFs were treated with CHX or DMSO, as a solvent control, 24 h before HCMV (TB40/E-GFP) infection at 1 TCID_50_/cell. RNA samples were collected at 24 hpi. **c** HFFs were infected with HCMV (TB40/E-GFP) at 1 TCID_50_/cell for 2 h and then media was changed for one containing GCV or DMSO as a solvent control. RNA samples were collected at 24 and 48 hpi. **d** Tetracycline-inducible IE1 and/or IE2 MRC-5 and ARPE-19 cells were treated with doxycycline. RNA samples were collected at indicated times. **a**–**d** RT-qPCR was performed using HSATII-specific primers. GAPDH was used as an internal control. *n* = 3. Data are presented as a fold change mean ± SD. **a**–**c** ***P < 0.001, ****P < 0.0001 by the unpaired, two-tailed *t*-test with (**b**, **c**) or without (**a**) Welch’s correction. ns—not significant. Open circles represent single data points

To determine if just viral attachment and/or entry with the introduction of virion contents to cells could trigger HSATII expression, we infected cells with replication-competent HCMV or replication-defective UV-irradiated virus. In comparison to cells receiving active virus, HSATII RNA induced by UV-irradiated virus was reduced by factors of ~1700 and ~100 at 24 and 48 hpi, respectively, as compared to its expression at 2 hpi (Fig. 2a). As a control, we showed that levels of a virion protein, pUL82 (pp71), increased following infection with replication-competent HCMV, but the tegument-delivered protein was degraded after infection with UV-irradiated virus with no new pUL82 accumulation (Supplementary Fig. 6). These data argue that active viral gene expression is necessary to induce HSATII expression. Cycloheximide (CHX) treatment strongly inhibited (33-fold reduction) HSATII accumulation compared to HCMV-infected cells treated with a solvent control (Fig. 2b), showing that de novo protein synthesis is needed to stimulate HSATII transcription. The viral DNA synthesis inhibitor, ganciclovir (GCV), which blocks the expression of late viral genes, did not change the HSATII levels at 24 or 48 hpi (Fig. 2c), arguing that immediate early (IE) and/or early (E) viral protein expression was sufficient to induce HSATII accumulation. As a control, accumulation of RNA from the late UL99 gene was assayed at 48 hpi, and, as expected, it was blocked by the drug (Supplementary Fig. 7).

Next, we focused on identifying which IE and/or E viral factor(s) were responsible for HSATII induction. We initially tested the viral IE1 and IE2 proteins, because they are known to be promiscuous transcriptional activators^13,17^. MRC5 fibroblasts and ARPE19 epithelial cells were prepared containing tetracycline-inducible IE1, IE2 or IE1+IE2 cDNAs, and western blot assays confirmed the induction of the viral proteins (Supplementary Fig. 8a). Although expression of IE1 or IE2 alone had little effect, expression of both proteins induced robust HSATII expression in fibroblasts and epithelial cells (Fig. 2d). The kinetics of HSAII expression was faster following induction of IE1+IE2-expression in epithelial cells than in fibroblasts, mimicking the difference evident in infected cells (Fig. 1c and Supplementary Fig. 2). The reason for the different kinetics between infected cell types is not clear. The magnitude of HSATII accumulation in our assays of IE1+IE2-expressing cells, although substantial, might be underestimated. IE1 from protein lysates of IE1+IE2-expressing cells migrated faster than the protein from infected cells when subjected to electrophoresis in an SDS-polyacrylamide gel (Supplementary Fig. 8b), suggesting IE1 produced outside the context of infection might lack one or more modifications. This could reduce IE1 transactivation, since posttranslational modifications are known to affect the activity of IE1 and IE2^18–20^. Further, the IE2 cDNA used to create IE2-inducible cells carries a single amino-acid substitution, A463T, which modestly reduces its transactivation activity compared to wild-type virus^21^. It is also possible that additional viral proteins contribute to the rate and extent of HSATII induction within infected cells. Nevertheless, IE1 and IE2 clearly act in concert to markedly induce the accumulation of HSATII transcripts from multiple chromosomal loci, as they are known to do for mRNA expression^13^.

### HSATII RNA modulates HCMV RNA, proteins, and progeny levels

**Fig. 3:**
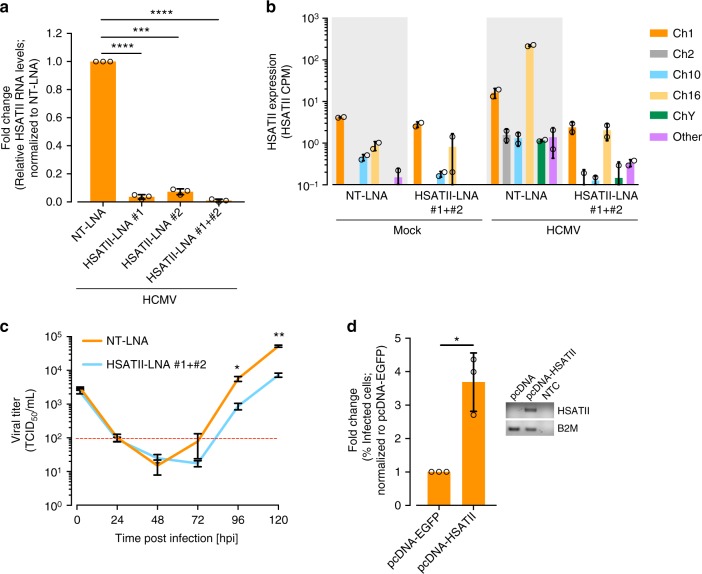
HSATII RNA is important for efficient HCMV yield. RNA samples were collected at 24 hpi from HFFs transfected with NT-LNA or HSATII-LNAs 24 h before HCMV (TB40/E-GFP) infection at 1 TCID_50_/cell. **a** RT-qPCR was performed using HSATII-specific primers. GAPDH was used as an internal control. Data are presented as a fold change mean ± SD. *n* = 3. ***P < 0.001, ****P < 0.0001 by the unpaired, two-tailed *t*-test with Welch’s correction. Open circles represent single data points. **b** RNA-seq analysis performed. Only unique HSATII reads were used to calculate its expression. HSATII expression in terms of CPM was computed and normalized across samples. *n* = 2. Open circles represent single data points. **c** Media samples were collected at indicated times from HFFs transfected with NT-LNA or HSATII-LNAs 24 h before HCMV (TB40/E-GFP) infection at 1 TCID_50_/cell. TCID50 ml^−1^ values were determined. *n* = 3. *P < 0.05, **P < 0.01 by the unpaired, two-tailed *t*-test. **d** Media samples were collected at 96 hpi from HFFs transfected with pcDNA or pcDNA-HSATII 48 h before HCMV (TB40/E-GFP) infection at 1 TCID_50_/cell. % of infected cells was calculated based on a number of IE1-positive cells in a reporter plate. Data are presented as a fold change mean ± SD. *n* = 3. *P < 0.05 by the unpaired, two-tailed *t*-test with Welch’s correction. Open circles represent single data points. Inside panel: RNA samples were collected from pcDNA or pcDNA-HSATII-transfected and HCMV-infected HFFs. HSATII-pcDNA primer set was used in RT-PCR analysis. B2M was used as an internal control. NTC non-template control sample

To investigate the possible biological role of HSATII during infection, we utilized locked nucleic acids (LNAs) that specifically target HSATII transcripts for degradation. The LNAs did not cause detectable non-specific cellular toxicity (Supplementary Fig. 9). Cells transfected with HSATII-specific LNAs (HSATII-LNAs) 24 h prior to infection had strongly decreased HSATII levels (Fig. 3a). RNA-seq analysis revealed that HSATII transcripts from all chromosomal loci in HCMV-infected cells were markedly decreased in HSATII-LNA-transfected cells compared with control NT-LNA transfected cells, but little effect on the low levels of HSATII RNAs was evident in mock-infected cells (Fig. 3b). Multiple cellular protein-coding transcripts were increased or decreased following LNA treatment, but no effect on coding RNA levels was evident in mock-infected cells (Supplementary Fig. 10a), arguing strongly against non-specific effects of the targeted LNAs. Additionally, HSATII-LNAs were very specific in downregulating HSATII versus other repeat RNAs (Supplementary Fig. 10b). Besides HSATII RNAs, which as a group were reduced by a factor of 90, only one simple repeat RNA [(AATGG)n] was reduced by a factor of five (Supplementary Fig. 10b). However, this result must be interpreted with caution. We detected only small number of simple repeat reads, and those reads might result from self-priming in the PCR amplification step of the RNA-seq protocol. Further, the simple repeat reads might be related to the expression of genes that have those repeats in their vicinity. Importantly, the (AATGG)n RNA was not induced by HCMV infection and its expression was not influenced by HSATII-LNAs in mock-infected cells.

We next investigated the effects of LNA-based HSATII knockdown on the production of extracellular HCMV progeny in fibroblasts. The ability of two individual HSATII-LNAs or their combination to efficiently decrease HSATII transcript levels (Fig. 3a) correlated with their effect on HCMV titer (Supplementary Fig. 11). With the use of both HSATII-LNAs together, HSATII knockdown reduced the accumulation of infectious virus at 96 and 120 hpi by a factor of ~8 as compared to controls when evaluated by TCID_50_ assay (Fig. 3c and Supplementary Fig. 11). Ectopically overexpressed HSATII RNA (Fig. 3d, inset) had the opposite effect, increasing the infectious yield by a factor of ~3.5× at 96 hpi (Fig. 3d). Together these data reveal that HSATII RNA supports the production of HCMV progeny.

As virus yield could be affected by perturbations at multiple steps of the viral replication cycle, we tested the effect of HSATII knockdown on levels of viral RNA, proteins and genomic DNA (vDNA) in infected cells. For RNA analysis, we quantified the expression of representatives from each of the three main classes of viral genes and HCMV long non-coding RNAs (lncRNAs). qRT-PCR determined HSATII suppression reduced levels of viral immediate-early (UL123, UL122, UL37x1), early (UL26, UL54), late (UL69, UL82, UL99), and lncRNAs (RNA4.9, RNA5.0 RNAs) at 96 hpi (Supplementary Fig. 12). The reduction for each of the tested RNAs was on the order of 70%. HCMV has a higher GC-content (~57%)^22^ than the cell, and viral coding RNAs can have CpG motif overrepresentation. However, those CpG motifs are not in a background of AU-rich sequences—as it is the case for HSATII sequences^11^, and are unlikely to react with HSATII-LNAs (Supplementary Fig. 13). Furthermore, RNA-seq analysis did not detect any significant effect of HSATII-LNAs on differential expression of HCMV transcripts at 24 hpi as compared to their expression in NT-LNA-treated cells (Supplementary Fig. 10c). Additionally, we did not find any correlation between the differential expression of HCMV transcripts at 24 hpi in NT-LNA- and HSATII-LNA-treated fibroblasts and the sequence similarity of the corresponding HCMV transcript sequences and HSATII-LNAs (Supplementary Fig. 14), which further advocates against off-target effects of the HSATII-LNAs directed toward HCMV transcripts. In sum, we conclude that the lower expression levels of multiple HCMV transcripts HSATII-deficient cells is related to lower HSATII levels in those cells and are not due to off-target effects of the HSATII-LNAs.

**Fig. 4:**
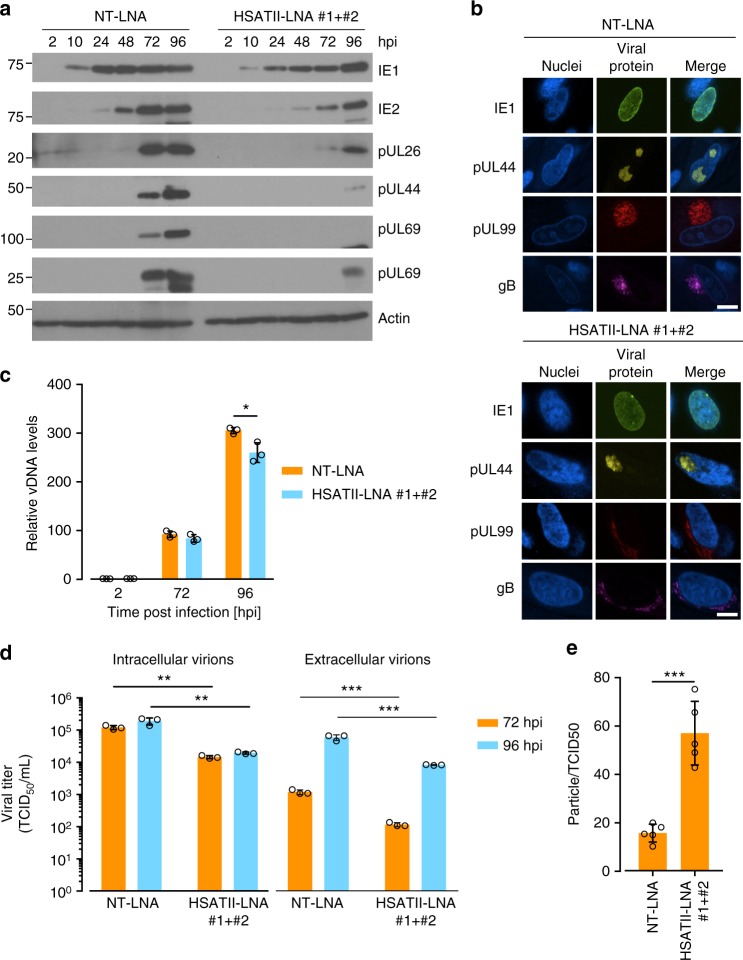
HSATII RNA is important for viral protein expression and localization, viral replication and release. **a** Protein samples were collected at indicated times from HFFs transfected with NT-LNA or HSATII-LNAs 24 h before HCMV (TB40/E-GFP) infection at 1 TCID_50_/cell. Protein levels were analyzed by the western blot technique using antibodies specific to IE1, IE2, UL26, pUL44, pUL69, and pp28. Actin was used as a loading control. **b** HFFs were transfected with NT-LNA or HSATII-LNAs 24 h before HCMV (TB40/E) infection at 1 TCID_50_/cell. At 72 hpi, cells were fixed and stained for IE1, ppUL44, p28 or gB and nuclei were counterstained with the Hoechst stain. Scale bar: 15 µm. **c** Total DNA was collected at indicated times from HFFs transfected with NT-LNA or HSATII-LNAs 24 h before HCMV (TB40/E-GFP) infection at 1 TCID_50_/cell. vDNA and cellular DNA copy numbers were determined. Data are presented as a fold change mean ± SD of the relative vDNA to cellular DNA ratio. *n* = 3. *P < 0.05 by the unpaired, two-tailed *t*-test. Open circles represent single data points. **d** Intracellular and extracellular virions were collected at indicated times from HFFs transfected with NT-LNA or HSATII-LNAs 24 h before HCMV (TB40/E-GFP) infection at 1 TCID_50_/cell. TCID50 ml^−1^ values were determined. Data are presented as a mean ± SD. *n* = 3. **P < 0.01, ***P < 0.001 by the unpaired, two-tailed *t*-test. Open circles represent single data points. **e** Particle-to-TCID_50_ ratios were calculated based on the TCID_50_ assay and vDNA copy numbers generated from media samples collected at 96 hpi from HFFs transfected with NT-LNA or HSATII-LNAs 24 h before HCMV (TB40/E-GFP) infection at 1 TCID_50_/cell. Data are presented as a particle-to-TCID_50_ ratio mean ± SD. *n* = 5. ***P < 0.001 by the unpaired, two-tailed *t*-test. Open circles represent single data points

Western blot assays indicated that the level of IE1 protein was reduced by a factor of 2–3 at each time point examined between 10 and 72 hpi in HSATII knockdown cells, but it reached the same level as in cells where HSATII was expressed normally by 96 hpi (Fig. 4a). In contrast, IE2 and the early and late viral proteins accumulated to significantly lower levels at each time tested in HSATII-deficient cells. The IE1 protein, which is spread throughout the nucleus, and the pUL44 subunit of the viral DNA polymerase, which accumulates in viral replication centers, were localized normally in HSATII-deficient cells (Fig. 4b). However, the late pp28 and gB virion proteins, which normally accumulate in the cytoplasmic assembly compartment, were partially mislocalized in infected cells lacking HSATII. A portion of each virion protein was spread through the larger part of cytoplasm (Fig. 4b and Supplementary Fig. 15). Thus, viral protein levels mimicked viral RNA levels, and portions of several late proteins were improperly localized. Consistent with perturbed viral protein expression and localization, HSATII knockdown reduced the level of intracellular vDNA to a limited extent (~20% reduction) at 96 hpi (Fig. 4c).

To further assess the effect of HSATII on virus production, we monitored the accumulation of intracellular and extracellular virus at 72 and 96 hpi. When HSATII RNA was knocked down, infectious virus was reduced in both locations by a factor of ~10 at both times after infection (Fig. 4d). As the viral titer represents not only the number of viral particles but also their infectivity, we determined the particle/TCID_50_ ratio for extracellular viral particles. By comparing DNase I-resistant vDNA to infectivity, we found that virions released from HSATII-deficient cells are less infectious (~2.5-fold) than those from control cells (Fig. 4e). As a control, we determined that our DNase treatment was effective in removing unprotected DNA (Supplementary Fig. 16). Although intracellular DNA was reduced to a limited extent, the number and infectivity of virions were reduced in the absence of HSATII RNA, presumably due to perturbations in the levels and localization of proteins that function during the late phase of infection.

### HSATII RNA alters cellular RNA levels and cell movement

**Fig. 5:**
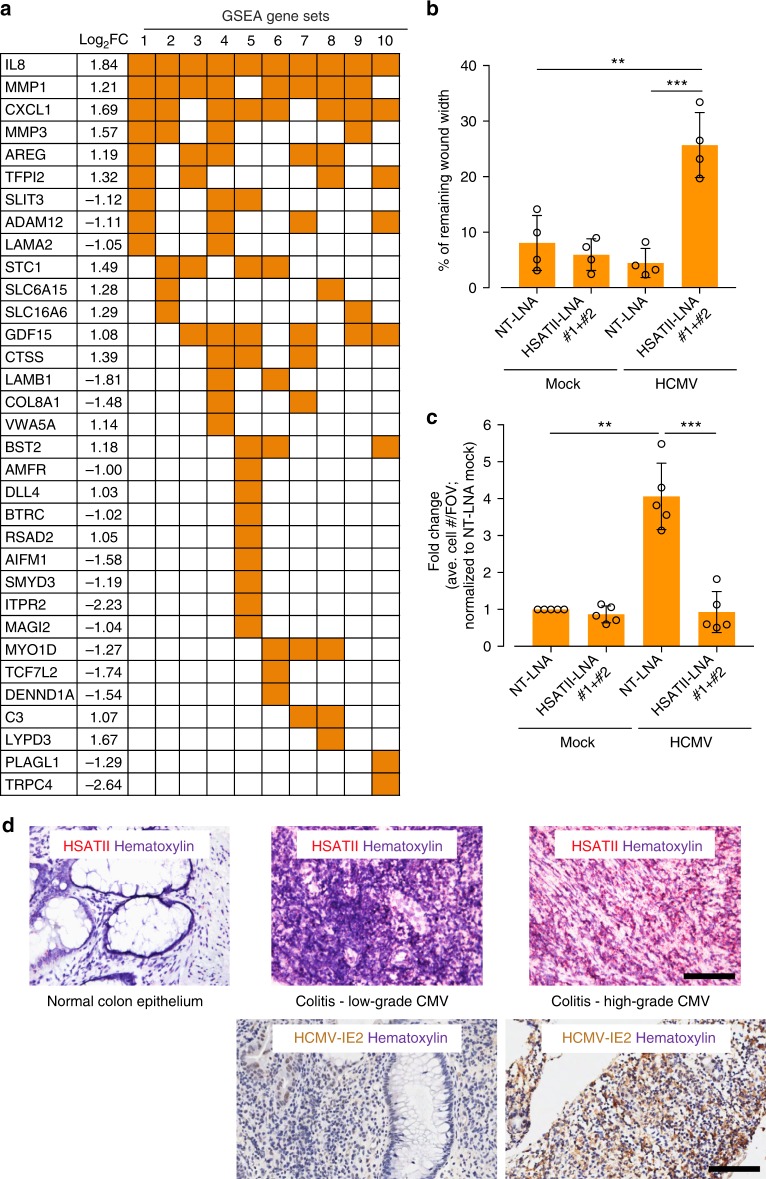
Role of HCMV-induced HSATII RNA in regulating cellular processes. **a** HSATII regulates expression of cellular genes. RNA samples were collected at 24 hpi from HFFs transfected with NT-LNA or HSATII-LNAs 24 h before mock or HCMV (TB40/E-GFP) infection at 1 TCID_50_/cell. RNA was isolated and analyzed using RNA-seq. GSEA was performed on the list of cellular genes differentially expressed in HCMV-infected, NT-LNA- versus HSATII-LNA-transfected HFFs. The matrix shows genes overlapping with specific gene set names (numbered) categorized based on increasing *P*-value and FDR *q*-value. GSEA-identified enriched gene sets: 1—HALLMARK EPITHELIAL MESENCHYMAL TRANSITION; 2—GHANDHI BYSTANDER IRRADIATION UP; 3—SATO SILENCED BY DEACETYLATION IN PANCREATIC CANCER; 4—GO CELLULAR RESPONSE TO ORGANIC SUBSTANCE; 5—NABA MATRISOME; 6—HAN SATB1 TARGETS DN; 7—DELYS THYROID CANCER UP; 8—ONDER CDH1 TARGETS 2 DN; 9—GHANDHI DIRECT IRRADIATION UP; 10—WANG SMARCE1 TARGETS DN. **b**, **c** HSATII regulates motility of epithelial cells. ARPE-19 cells were transfected with NT-LNA or HSATII-LNAs 24 h before mock or HCMV (TB40-epi) infection at 1 TCID_50_/cell. After 2 hpi, wound was created and its closure was monitored. The graph shows a wound closure at 44 hpi. Data from biological replicates are presented as a percent of remaining wound width mean ± SD. *n* = 4. **P < 0.01, ***P < 0.001 by the unpaired, two-tailed *t*-test. Open circles represent single data points. **c** ARPE-19 cells were transfected with NT-LNA or HSATII-LNAs 24 h before mock or HCMV (TB40-epi) infection at 1 TCID_50_/cell. After 6 hpi, cells were transferred onto transwell inserts. Migrated cells were washed, fixed, and nuclei stained. The graph presents a fold change mean ± SD based on a number of cells migrated through a transwell per FOV. Data from biological replicates are presented as a fold change mean ± SD. *n* = 5. **P < 0.01, ***P < 0.001 by the unpaired, two-tailed *t*-test with Welch’s correction. Open circles represent single data points. **d** HSATII is markedly elevated in HCMV colitis. Paraffin-embedded sections of normal epithelium, low HCMV antigen-positive, or high HCMV antigen-positive CMV colitis sections were processed. HSATII RNA was visualized by ISH assay using HSATII-specific probe. An intensely brown stain characterizes CMV antigen-positive cells. Nuclei were counterstained with hematoxylin (purple stain) and HSATII is shown as red stain. Scale bar: 100 µm

To investigate the effect of HSATII on infected-cell biology, we utilized RNA-seq to monitor global gene expression of cells treated with control or HSATII-LNAs. No effect of LNA treatment was evident in mock-infected cells; in contrast, the levels of multiple cellular coding RNAs were modulated within infected cells (Supplementary Fig. 10a). IPA and GSEA analyses of differentially regulated RNAs strongly associated virus-induced HSATII RNA with the regulation of protein stability and posttranslational modifications, and particularly with cellular movement (Fig. 5a and Supplementary Fig. 17). Cells treated with HSATII-LNAs exhibited decreased expression of RNAs including ADAM12, TCF7L2, PLAGL1, SLIT3, DIO2, and LPP, as well as increased levels of CXCL1, CXCL8, MMP1, MMP3, STC1, and CTSS (Fig. 5a). Of note, the latter genes are associated with inflammation and oncogenesis^23^, thus our results provide additional support for a role of HSATII RNA in immune regulation and cancer progression as discussed above for tumor cells.

As noted above, reduced HSATII RNA levels in infected cells modulated the expression of genes associated with cell movement. HCMV is known to modulate the motility of multiple cell types^24–28^, a phenotype with the potential to influence both HCMV spread and latency within its infected host^24,29^. Since HCMV triggers high levels of HSATII RNA in epithelial cells (Fig. 1a and Supplementary Fig. 2), a cell type playing an important role in HCMV pathogenesis^30^ and the wound healing process^31^, we asked if modulating HSATII RNA levels would lead to changes in wound closure or migration of infected epithelial cells. A wound-healing assay^32^ revealed that HCMV-infected cells lacking high HSATII levels were much slower in closing wounds compared to uninfected cells, and this effect was even more pronounced when compared to infected cells with normal, high levels of HSATII RNA (Fig. 5b). A transwell migration assay confirmed that cells characterized by a low HSATII RNA level were also less mobile (~4×) than HCMV-infected cells with a highly induced HSATII expression (Fig. 5c). Our preliminary data showed that transfection efficiency for exogenous expression of HSATII was too low in epithelial cells preventing the assessment of results from the wound healing and transwell migration assays. Overall, our experimental results are consistent with the possibility that HSATII induction promotes a transcriptional environment permissive for cell movement.

### HSATII RNA is elevated in CMV colitis

A hallmark of severe HCMV infection is the involvement of multiple organs^33^. Infection of the gastrointestinal tract may lead to the onset of CMV colitis, which in rare cases of immunocompetent individuals resembles gastroenteritis and in patients with a compromised immune system is the second most frequent outcome of CMV disease after CMV retinitis^34,35^. We used RNA ISH^4^ to evaluate the levels of HSATII RNA in normal colon epithelium versus tissue biopsies from two patients manifesting low or high grade of CMV colitis. Low versus high grade was based on a standard immunohistochemical (IHC) assay staining IE and E CMV antigens (CCH2-UL44/DDG9-IE)^36,37^ or the IHC assay specifically staining HCMV IE2 protein (Fig. 5d and Supplementary Fig. 18). We noticed that the latter staining method seemed to be more sensitive (Fig. 5d and Supplementary Fig. 18). To our knowledge, IHC staining of colitis samples with the use of our IE2 antibodies^38^ is a novel approach and was utilized after we determined that HCMV IE1 and IE2 proteins work cooperatively in inducing HSATII RNA (Fig. 2d). At this point, we can only speculate that IE2 antibody might possess better binding characteristics for IHC staining in our tested samples. As with uninfected fibroblasts (Fig. 1e), normal colon epithelium was negative for HSATII-specific signal (Fig. 5d). We observed a concordance between the level of CMV infection based on detection of viral proteins by IHC and the strength of the HSATII RNA signal (Fig. 5d and Supplementary Fig. 18).

Identifying patients with CMV colitis is rare given the challenging diagnosis. Our very limited patient population prevents us from making a strong conclusion about HSATII involvement in the pathogenesis of CMV colitis. Nevertheless, as we determined that HCMV IE1 and IE2 proteins cooperate to induce HSATII expression (Fig. 2d), the positive staining for IE2 protein in colitis samples is consistent with the possibility that elevated levels of HSATII could result from regulation by viral proteins in this tissue as well. Moreover, these results lead us to speculate that elevated HSATII RNA may either directly affect the development of CMV colitis or influence the disease by augmenting HCMV replication in the tissue. More work is needed to address these intriguing questions, but to our knowledge, our report is the first documentation of elevated HSATII RNA in likely virally infected tissue.

## Discussion

There are a number of striking similarities between chronic virus-infected cells and cancerous cells. These include manipulative and evasive interactions with the innate and adaptive immune system, metabolic changes and changes in cell division to provide substrates for replication, epigenetic alterations in cells to promote viral replication or cancer cell spreading, and extensive communication between cells and tissues. The induction of HSATII RNA synthesis in virus-infected cells and many cancers appears to utilize all of these altered cellular processes^1,4,5,9^ for the benefit of the fitness of the cancer cells or the virus. HSATII RNA can affect the innate immune system inducing the synthesis of IL-6 and TNF-alpha^11^. HSATII RNA and some viruses (i.e., Avian Influenza A) share RNA nucleotide motifs that appear to be recognized by components of the innate immune system (such as ZAP^12,39^) or pattern recognition receptors^11^ and this can result in evolutionary selection pressures that change the viral genome sequences with time and replication^10,40^. The results presented here document the role of HSATII in cellular motility that could enhance the fitness of both the virus and cancer cells in the environment of the host.

CpG motifs in HIV1 RNA have negative effects on viral infection^12^. In contrast, HCMV, another chronic human virus implicated in oncomodulation^41–43^, induces endogenous repeated CpG-rich transcripts that mimic those found in zoonotic, non-mammalian viruses. As previous work argues that HSATII expression provides a positive fitness advantage for tumors^9,15,16^, we believe both cancers and chronic DNA viruses are specifically utilizing this RNA species to their selective advantage. A tumor upregulating HSATII would therefore be engaging in genuine viral mimicry by evolving to induce specific features of viruses foreign to humans to gain an evolutionary advantage, as opposed to inducing non-specific generic features of RNA viruses, such as long dsRNA, to its disadvantage as a possible consequence of epigenetic stress. The shared ability of at least some herpesviruses and cancers to utilize this particular RNA repeat is an intriguing example of convergent evolution mechanism in these seemingly disparate biological settings. As our study has not found the virus-induced HSATII expression beyond herpesvirus family, it is intriguing to speculate that our mechanistic work, documenting HCMV IE1 and IE2 proteins working cooperatively to induce high HSATII RNA levels in fibroblasts and epithelial cells together with the IE2 staining seen in CMV colitis biopsies, may provide another regulatory layer to the processes important in pathology of diseases associated with some herpesviruses, such as colitis, retinitis, encephalitis, pneumonia, hepatitis, cancer^44,45^, and diseases (i.e., Alzheimer’s disease) recently linked to herpesvirus infection^46–48^. As an increasing body of evidence suggests HCMV could be categorized as an oncomodulatory virus^42,49,50^, we hypothesize virus-induced HSATII as an oncomodulatory factor. Our observations provide a new framework—viral infection—for further investigation of the role of this family of non-coding RNAs in human inflammatory disease.

## Methods

### Ethics statement

Colon samples were analyzed under a Partners/MGH IRB approved protocol 2016P002541. This is a discarded tissue protocol with minimal risk to patients.

### Cells, viruses, and reagents

Human lung fibroblasts (MRC-5), human dermal fibroblasts (HDF; immortalized by expressing SV40 large T antigen), and human retinal pigment epithelial (ARPE-19) cells^51^ were from the American Type Culture Collection (ATCC). HCV-infected Huh7.5 cells (A gift of Dr. Charles M. Rice, Rockefeller University) are from Ploss lab (Princeton University). Primary HFFs and other fibroblasts were cultured in Dulbecco’s Modified Eagles Medium (DMEM) supplemented with 10% fetal bovine serum (10% FBS/DMEM) (Sigma-Aldrich, St. Louis, MO). HFFs were used at passages 8–13. ARPE19 cells were cultured with added Ham’s F-12 nutrient mixture (Sigma-Aldrich). 100 units ml^−1^ of penicillin (Sigma-Aldrich) and 95 µg ml^−1^ of streptomycin (Thermo Fisher Scientific, Waltham, MA) were added to media.

To construct IE1 and IE2 expressing cell lines, cDNAs encoding 72 kDa IE1 (IE-72) and 86 kDa IE2 (IE-86) from strain Towne were PCR amplified from pLXSN-IE1^52^ and pLXSN-IE2, respectively. The IE2 cDNA contains missense mutations at methionine 242 (M242I), eliminating the internal start responsible for generating the 40 kDa IE2-40 protein^53^, and alanine 463 (A463T), which reduces IE2’s transactivation activity by about 50%^21^. A cDNA of monomeric EGFP was subcloned from a derivative of pEGFP-N3 (Clonetech) containing the mutation A206K^54^. Tetracycline-inducible cell lines expressing IE1, IE2, or EGFP were created by inserting each cDNA into pLVX-TetOne-Puro (Clonetech), producing VSV-G pseudotyped lentivirus particles in 293FT cells, concentrating lentivirus particles by ultracentrifugation over a 20% sorbitol cushion, and transducing MRC-5 or ARPE-19 cells. Stable cell lines were selected for 1 week in the presence of puromycin. Dual IE1 and IE2 expressing cells were created by cloning Towne IE2 into a derivative of pTetOne-Puro where the endogenous SV40-promoter-puromycin cassette was removed and a porcine teshovirus 2A-Neomycin geneblock (P2A-Neomycin) was inserted on the 3-prime-end of the reverse-Tetracycline transactivator (rtTA). Lentivirus particles were prepared as above. Stable lines were generated by co-transducing IE1 and IE2 lentivirus particles and selecting for 1 week in the presence of puromycin and G418.

Two GFP-tagged viruses derived from clinical isolates, TB40/E-GFP^55,56^, FIX-GFP^57^, a GFP-tagged laboratory strain AD169-GFP^58^, as well as untagged TB40/E were used in these studies. TB40-epi designates the TB40/E strain grown in ARPE-19 cells^59^. Viruses were produced from BAC clones transfected with pp71 expression plasmid into HFFs, MRC-5, or ARPE-19 cells to generate viral progeny of wild-type growth characteristics^58^. Viruses were purified by centrifugation through a sorbitol cushion (20% sorbitol, 50 mM Tris–HCl,1 mM MgCl_2_, pH 7.2), concentrated and resuspended in DMEM. Viral titers were determined using a tissue culture infectious dose 50 (TCID_50_) assay on HFFs or ARPE-19 cells, and infections were performed at a multiplicity of 3 TCID_50_/cell or as designated. UV-inactivation of TB40/E-GFP virions was performed by 4 sequential UV irradiations of viral inoculum using Auto Cross Link settings (UV Stratalinker 2400; San Diego, CA).

HSV-1 strain F^60^ was kindly provided by B. Roizman (University of Chicago) and grown in Vero cells. Pooling cell-associated virus, obtained by sonication, with cell-free virus, produced viral stocks. HSV-1 titers were determined using TCID_50_ assay. Fibroblasts were infected with HSV1 at a multiplicity 3 TCID_50_/cell. Adenovirus (Ad5) was kindly provided by S. J. Flint (Princeton University). Ad5 titer was determined on MRC-5 cells by a focus forming assay and is expressed as focus forming units (FFU). Fibroblasts were infected with Ad5 at a multiplicity 10 FFU/cell. Influenza A virus [IAV; A/PR/8/1934(H1N1) (ATCC)] titer was determined using TCID_50_ assay. HFFs were infected with IAV at a multiplicity 3 TCID_50_/cell in Flu infection buffer [DMEM containing 0.2% BSA, 1 μg/ml L-1-tosylamido-2-phenylethyl chloromethyl ketone (TPCK)-treated trypsin (Thermo Fisher Scientific) and 0.1% FBS]. Zika virus (ZIKV; ZIKV/1947/UG/MR766) titer was determined using a plaque assay. HDFs were infected with ZIKV at a multiplicity 10 PFU/cell. Hepatitis C Virus (HCV; JCI strain expressing Cre recombinase) titer was determined on Huh-7.5 cells using TCID_50_ assay. Huh-7.5 cells were infected with HCV at a multiplicity 1 TCID_50_/cell.

Following a 2-h absorption period for all viruses, inoculum was removed, cells were washed twice with complete medium, and collected at indicated time points post infection. When indicated, experimental HCMV viral titers were also determined by assaying for IE1-positive cells on reporter plates^61^.

To measure the portion of cells within a culture that were infected, fibroblasts were fixed with methanol and stained using mouse antibodies anti-HCMV IE1 (1B12; 1:100 dilution), anti-HSV ICP4 (1:10 dilution)^62^, anti-Ad5 E2 (DBP; B6; 1:50 dilution)^63^, anti-IAV nucleoprotein (HB-65; 1:50 dilution), or anti-Flavivirus Group Antigen Antibody (D1-4G2-4-15; 1:10 dilution; Sigma, cat.# MAB10216) and goat anti-mouse Alexa Fluor-488 conjugated secondary antibody (1:1000 dilution; Invitrogen, cat.# A11029). Nuclei were counterstained with Hoechst 33342. Cells were visualized and the percentage of viral antigen-positive cells was calculated from at least 20 fields of view using the Operetta high-content imaging and analysis system (PerkinElmer).

Cyclohexamide (Sigma-Aldrich) and ganciclovir (Sigma-Aldrich) were dissolved in DMSO and used at 100 μg ml^−1^ or 50 µM concentrations, respectively. Doxycycline (Sigma-Aldrich) was dissolved in water and used at 2 μg ml^−1^. Puromycin was dissolved in water and used at 1.5 µg ml^−1^ (MRC-5) or 2 µg ml^−1^ (ARPE-19). G418 was dissolved in water and used at 800 µg ml^−1^ (MRC-5) or 1 mg ml^−1^ G418 (ARPE-19).

### RNA analysis

For RNA sequencing (RNA-seq) analysis, RNA from HCMV-, HSV1-, or Ad5-infected cells at defined multiplicities of infectious units/cell and appropriate mock-infected cells was collected in QIAzol Lysis Reagent (Qiagen) at 48, 9, or 24 hpi, respectively. The specific times of sample collection were chosen to capture the viral replication cycles at their halfway points. RNA was isolated using the miRNeasy Mini Kit (Qiagen). DNA was removed from samples using Turbo DNase (Thermo Fisher Scientific) and RNA quality was analyzed using the Bioanalyzer 2100 (Agilent Technologies, Santa Clara, CA). cDNA sequencing libraries were prepared by the Penn State College of Medicine Genome Sciences Facility using the TruSeq Stranded Total RNA with Ribo-Zero kit (Illumina, San Diego, CA) for rRNA depletion, and subjected to multiplexed sequencing (RNA-seq) using Rapid HiSeq2500 sequencer (Illumina) for 100 cycles in paired-end, rapid mode (2 × 100 bp).

RNA-seq data was de-multiplexed based on indexes and raw Illumina reads were quality filtered as follows. First, ends of the reads were trimmed to remove N’s and bases with quality less than 20. After that the quality scores of the remaining bases were sorted and the quality at the 20th percentile was computed. If the quality at the 20th percentile was less than 15, the whole read was discarded. Also, reads shorter than 40 bases after trimming were discarded. If at least one of the reads in the pair failed the quality check and had to be discarded, we discarded the mate as well. Human, HCMV, HSV1, and Ad5 fasta and annotation (.gtf) files were created for mapping by combining sequences and annotations from Ensembl annotation, build 37, repbase elements (release 19) and TB40/E (EF999921.1), FIX (GU179289), AD169 (FJ5275630), HSV1 (GU734771), or Ad5 (AC000008) when appropriate. To that created concatenated human-virus genomes, quality filtered reads were mapped using STAR aligner^64^. Aligned reads were assigned to genes using the featureCounts function of Rsubread package^65^ with the external Ensembl annotations^66^. This produced the raw read counts for each gene. Gene expression in terms of log2-CPM (counts per million reads) was computed and normalized across samples using the trimmed mean of M-values method (TMM)^67^, as implemented in the calcNormFactors function of edgeR package^68^. Differential expression analysis was performed using limma package^69,70^. Expression data were used in conjunction with the weights computed by the voom transformation^71^.

To calculate the percent of HSATII reads originating from each chromosome in infected cells and in selected samples from the Cancer Genome Atlas (TCGA), we identified uniquely mapped reads that exclusively overlapped with HSATII repeat. The number of normalized counts of HSATII reads mapped to each chromosome was computed. Next, the percentage of these reads mapping to each chromosome was calculated by dividing their number by the total number of HSATII reads and multiplying by 100%. We only considered samples with at least 100 HSATII reads. TCGA samples were comprised of 12 LUAD (lung adenocarcinoma), 10 COAD (colon adenocarcinoma), 5 BRCA (breast invasive carcinoma), 4 KIRC (kidney renal clear cell carcinoma), 4 UCEC (uterine corpus endometrial carcinoma), and 3 BLCA (bladder urothelial carcinoma) tumors.

Gene Set Enrichment Analysis (GSEA) was also used to investigate the data set overlap with annotated gene sets comprising the Molecular Signature Database (MSigDB). A matrix of differentially expressed genes from the data set significantly matching identified MSigDB gene sets were composed and ordered based on a number of overlapping genes, *P* value determining the probability of association with a given gene set and a false discovery rate *q*-value.

For quantitative reverse transcription PCR (qRT-PCR) analysis, cells were collected in QIAzol Lysis Reagent (Qiagen). To fractionate RNA, DNA, and proteins, chloroform was added; samples were spun at 12,000×*g* for 15 min at 4 °C. RNA from an aqueous layer was isolated using the miRNeasy Mini kit (QIAGEN) according to the manufacturer’s instructions. RNA samples were stored at −80 °C. DNA contaminants were removed from the samples using the TURBO® DNase Kit (Invitrogen by Thermo Fisher Scientific) according to the manufacturer’s instructions. cDNA was made using random hexamers (Invitrogen by Thermo Fisher Scientific) and Superscript™III Reverse Transcriptase Kit (Invitrogen by Thermo Fisher Scientific) according to the manufacturer’s instructions. Quantitative PCR (qPCR) was performed using SYBR Green master mix (Applied Biosystems by Thermo Fisher Scientific, Foster City, CA) on the QuantStudio 6 Flex-Real Time PCR System (Applied Biosystems by Thermo Fisher Scientific). For a semiquantitative PCR, product amplification was carried out using PTC-225 thermocycler (MJ Research Inc., BioRad Laboratories), with the following PCR mix: 10× PCR Reaction Buffer with MgCl_2_ (Roche), 1.25 units of Taq DNA Polymerase (Roche) and a 200 μM concentration of each deoxynucleotide (Thermo Fisher Scientific). The performance of HSATII-specific primer sets was tested for uniformity and consistency across serially diluted cDNA sample and show a high level of linearity during amplification (Supplementary Fig. 19a–e).

Primer sequences used in qRT-PCR reactions are listed in Supplementary Table 1. Transcript levels were analyzed using the ∆∆Ct method and GAPDH or B2M were used as an internal control^72^. Data are presented as a fold change mean ± SD. The unpaired, two-tailed *t*-tests were performed and *P* value was used to measure a statistical significance between samples.

### Protein analysis

Cells were either harvested using protein lysis buffer [50 mM Tris–HCl at pH 7.5 (Thermo Fisher Scientific), 5 mM ethylenediaminetetraacetic acid (EDTA; Thermo Fisher Scientific), 100 mM sodium chloride (Thermo Fisher Scientific), 1% Triton X-100 (Thermo Fisher Scientific), 0.1% sodium dodecyl sulfate (SDS; Roche), and 10% glycerol (Sigma)] or Trizol. If Trizol was used, upon RNA/DNA/protein fractionation and the removal of RNA and DNA fractions, proteins were precipitated by adding 2-propanol. After pelleting proteins at 12,000 × *g* for 10 min at 4 °C, the pellet was washed with of 0.3 M GuHCl/95% EtOH, washed with 100% EtOH, resuspended in 1:1 1% SDS:8 M urea/1 M tris(hydroxymethyl) aminomethane (Tris) and sonicated. Protein samples were stored at −80 °C. Protein samples were mixed with 6× SDS sample buffer (325 mM Tris pH 6.8, 6% SDS, 48% glycerol, 0.03% bromophenol blue) containing 9% 2-mercaptoethanol (Sigma). Proteins were separated by electrophoresis (SDS-PAGE) and transferred to ImmunoBlot polyvinylidene difluoride (PVDF) membranes (BioRad Laboratories). Western blot analyses were performed using mouse monoclonal antibodies anti-IE1 (1B12; 1:500 dilution)^73^, anti-IE2 (3A9; 1:500 dilution), anti-pUL26 (7H1-5; 1:100 dilution)^74^, pUL44 (CMV ICP36; 1:80,000 dilution; Virusys; Taneytown, MD; cat.# CA006), anti-pUL69 (10E11; 1:100 dilution)^75^, anti-pUL82 (10G11; 1:100 dilution)^76^, anti-pUL99 (10B4-29; 1:100 dilution), anti-GFP (1:1400 dilution; Sigma; cat.# 11814460001) and anti-β-actin-HRP (1:100,000 dilution; Abcam; cat.# ab49900). Goat anti-mouse antibody (1:10,000 dilution; Jackson ImmunoResearch Laboratories Inc.; cat.# 115-035-003) conjugated with horseradish peroxidase was used as secondary antibodies. Western blots were developed using WesternSure ECL Detection Reagents (Licor). The uncropped images of western blots are shown in the Supplementary Fig. 20.

### Immunofluorescence

Infected cells were fixed and stained for viral proteins: HCMV (IE1, ppUL44, p28 or gB), HSV1 (ICP4), Ad5 (DBP), IAV (NP) or ZIKV (the flavivirus antigen) and nuclei were counterstained with the Hoechst stain. Cells were visualized using either Nikon Ti-E confocal microscope (Nikon Instruments Inc.) with spinning disc, 30× or 60× magnification and Z-stack mode, or the Operetta high-content imaging system (PerkinElmer; Waltham, MA) with 20× magnification.

### DNA analysis

Cells were harvested and DNA was isolated using the DNA Blood & Tissue Kit (Qiagen). Intracellular viral DNA was quantified from total intracellular DNA. Extracellular viral DNA was isolated from sample media collected at 96 hpi. Media was treated with 30 units of DNase I (Invitrogen by Thermo Fisher Scientific, Carlsbad, CA) according to the manufacturer’s recommendations. Virions in the media were lysed and isolated using the DNA Mini Kit (QIAGEN, Hilden, Germany) according to the manufacturer’s instructions.

vDNA and cellular DNA copy numbers were determined based on standard curves of viral genomic UL44 (Forward: 5′-GTGCGCGCCCGATTTCAATATG-3′, Reverse: 5′-GCTTTCGCGCACAATGTCTTGG-3′) or cellular genomic GAPDH (Forward: 5′-CCCCACACACATGCACTTACC-3′, Reverse: 5′-CCTAGTCCCAGGGCTTTGATT-3′) amplified from serially diluted HCMV TB40-BAC4 DNA or pUC18-gGAPDH DNA, respectively. Data are presented as a fold change mean ± SD. Student’s *t*-test was performed and *P* value was used to measure a statistical significance between samples.

### HSATII RNA knockdown

Locked nucleic acid (LNA™; Exiqon, Skelstedt, Denmark) oligonucleotides were designed to target identified, highly abundant HSATII transcripts from different chromosomal loci. The most effective LNA™s: HSATII-LNA #1 (5′-CCATTCGATAATTCCG-3′), HSATII-LNA #2 (5′-GATTCCATTCGATGAT-3′), or a mixture of both HSATII-LNAs (#1 + #2) were used for experiments as indicated. Lipofectamine RNAiMAX® Reagent (Thermo Fisher Scientific, Waltham, MA) and LNAs were resuspended in Opti-MEM® medium (Thermo Fisher Scientific) according to the manufacturer’s instructions. The final LNA concentration applied to cells was 100–200 nM. Non-target scrambled sequence LNA (NT-LNA; 5′-AACACGTCTATACGC-3′) was used as a negative control. HFFs and ARPE-19 cells were incubated for 24 h before being mock- or HCMV-infected. Cells were collected at the indicated time post-infection using QIAzol buffer (QIAGEN, Hilden, Germany) and stored at −80 °C until sample processing.

### Plasmid transfection

HFFs at 70% confluency were transfected with 1 µg of pcDNA3.1 (Addgene) or pcDNA-HSATII (a generous gift of Arnold Levine) using X-tremeGENE 9 DNA Transfection Reagent (Roche) according to the manufacturer’s instructions. 24 h later, plasmid-transfected cells were infected with TB40/E-GFP at a multiplicity of 3 TCID_50_/cell. Media and RNA samples were collected at 96 hpi and stored at −80 °C.

### Cell migration assays

To perform wound healing assays, confluent monolayers of NT-LNA- or HSATII-LNA-transfected ARPE-19 cells were infected with TB40-epi at a multiplicity of 3 TCID_50_/cell or were mock infected. At 2 hpi, cells were washed to remove inoculum and scratching the cell monolayer with 1-ml pipet created wounds. The process of wound closure was monitored in time and pictures of wounds were taken using the Nikon Eclipse TE2000-U inverted microscope. The average wound width (in arbitrary units) of ARPE-19 cells was calculated from the captured images using ImageJ software^77^. Results are plotted as a percent of remaining wound width mean ± SD.

To perform transwell migration assay, NT-LNA- or HSATII-LNA-transfected ARPE-19 cells were infected with TB40-epi at a multiplicity of 3 TCID_50_/cell or mock-infected. At 6 hpi, cells were trypsinized and 5 × 10^4^ cells were seeded onto each filter in FBS-free medium containing ITS Liquid Media Supplement (Sigma-Aldrich). After 24 h at 37 °C/5% CO_2_, filters were washed with 1× PBS and fixed in methanol. Non-migrated cells were removed with a cotton swab, and nuclei of migrated cells on the bottom surface of the filter were stained with Hoechst 33342 and were imaged by the Nikon Eclipse TE2000-U inverted microscope. Migrated cell number was quantified from the captured images using ImageJ software^77^. Results are plotted as a fold change mean ± SD of average cell number per field of view (FOV).

### RNA in situ hybridization (ISH) assay

To analyze HSATII levels in HCMV-infected cells, HFFs were infected with HCMV at a multiplicity of 1 TCID_50_/cell or mock-infected. At 24 hpi, cells were collected, washed with 1× PBS and resuspended in human plasma (Sigma-Aldrich). To facilitate sample coagulation, 13 NIH units of thrombin (Sigma-Aldrich) were added to each sample. Cells were then fixed in 10% formaldehyde for 4 h. The fixed pellets were transferred to biopsy cassettes. Automated ISH assays for HSATII RNA was performed using the ViewRNA eZ-L Detection Kit (Affymetrix by Thermo Fisher Scientific) on the BOND RX IHC and ISH Staining System with BDZ 6.0 software (Leica Biosystems Inc., Buffalo Grove, IL). Cell pellets were formalin-fixed and paraffin-embedded (FFPE) and cut in 5-µm sections on slides and processed automatically from deparaffinization, through ISH staining and hematoxylin counterstaining. Automatic coverslipper (Leica Biosystems) was used for coverslipping slides. Briefly, slides were baked for 1 h at 60 °C, and placed on the BOND RX for processing. The BOND RX user-selectable settings were the ViewRNA ez-L Detection 1-plex (Red) protocol and ViewRNA Dewax1; ViewRNA HIER2 (90) 5 min; ViewRNA Enzyme 2 (5 min); ViewRNA Probe Hybridization 3 h. With these settings, the RNA unmasking conditions for the tissue consisted of a 5-min incubation at 90 °C in Bond Epitope Retrieval Solution 2 (Leica Biosystems) followed by 5-min incubation with Proteinase K from the BOND Enzyme Pretreatment Kit at 1:1000 dilution (Leica Biosystems). The HSATII RNA-targeting Probe (Affymetrix; Cat #VA1-10874; probe sequence: ATCATCGAATGGAATCGAATG) was diluted 1:40 in ViewRNA Probe Diluent (Affymetrix) for use on the automated platform. Diluted Probe Set, diluted Proteinase K, and ViewRNA eZ-L Detection Kit were loaded onto BOND RX prior to starting the run. After the run, post rinsing with water and drying for 30 min at room temperature, slides were dipped in xylene, and mounted using HistoMount solution (Life Technologies by Thermo Fisher Scientific). HSATII signal from ISH experiments was quantified based on the ratio of HSATII signal area to cell area using BDZ 6.0 software.

To analyze HSATII levels in human biopsies of HCMV colitis, normal colon and two CMV positive colitis biopsies were analyzed. It is of note that identifying these patients is complicated and rare given the difficulty in the diagnosis of CMV colitis. Both patients had ulcerative colitis on immunosuppressive medications predisposing them to CMV infection. The diagnosis was made with biopsy of the colon and immunohistochemistry analysis performed by a board-certified anatomic pathologist. Immunohistochemical expression of the CMV was evaluated by deparaffinizing FFPE sections by baking them for 1 h at 60 °C. IHC staining was done on the BondRx using the BOND Polymer Refine Detection kit (Catalogue No. DS9800). Antigen retrieval was carried out with citrate buffer at pH 6 for 10 min using Bond Epitope Retrieval Solution 1 (Leica Biosystems). Mouse monoclonal antibodies against HCMV (antibody mixture to infected cell lysate, clone CCH2+DDG9, Sigma-Aldrich); HCMV IE2 (clone 3H9)^78^ were diluted in Bond^TM^ Primary Antibody Diluent (Leica Biosystems Inc.) and signal was detected by the Polymer Refine Kit (Leica Biosystems Inc.) and protocol F on a Leica Bond Rx Autostainer. Automated ISH assay for HSATII RNA was performed as described for HCMV-infected fibroblasts.

### Statistical analysis

To determine statistical significance among samples in experiments, unpaired, two-tailed *t*-tests were performed between the arrays of data from distinct samples to determine *P* values. *P* value < 0.05 was considered significant. The significance is shown by the presence of asterisks above data points with one, two, three or four asterisks representing *P* < 0.05, *P* < 0.01, *P* < 0.001, or *P* < 0.0001, respectively. Only significant *P* values are reported.

### Reporting summary

Further information on experimental design is available in the Nature Research Reporting Summary linked to this article.

## Supplementary information

Supplementary Information

Reporting Summary

## Data Availability

Raw RNA-seq data is available from NCBI Gene Expression Omnibus (GEO) under accession numbers GSE120890 and GSE120891. All relevant data are available from the authors.
