# Supplementary material for: A tumor-specific endogenous repetitive element is induced by herpesviruses
Source: Nat Commun. 2019 Jan 9;10:90. doi: 10.1038/s41467-018-07944-x (PMC6327058; doi:10.1038/s41467-018-07944-x)
Supplement: Supplementary file 1 — Supplementary Information [file 41467_2018_7944_MOESM1_ESM.pdf]

Supplementary Information for:

A Tumor-Specific Endogenous Repetitive Element Is Induced by Herpesviruses

Nogalski et al.

Including:

Supplementary Methods

Supplementary Figures 1-20 with corresponding Figure Legends

Supplementary Table 1

Supplementary References

SUPPLEMENTARY METHODS

RNA analysis

Ingenuity Pathway Analysis (IPA) cloud software (Qiagen) was used to overlay differentially expressed genes onto global molecular network information incorporated in the Ingenuity Pathway Knowledge Base. The Core Analysis in IPA was used to organize our data set into gene ontologies and to identify predicted biological functions and processes relevant to the data set based on *P* value determining the probability of association with a given gene set.

CpG bias of the viral genes and contiguous 500bp segments of the viral genome was computed using statistical methods developed by Greenbaum et al.[1]

The best local alignment of LNAs to each of the viral genes was identified using water program of EMBOSS package[2] with gap opening and gap extension penalties set to 10 and default score matrix. The best alignment score for each gene was plotted against $\log_2$(fold change of gene expression) between HCMV-infected cells treated with NT-LNA or HSATII-LNA #1+#2. The maximal score was chosen out of the score for LNA #1 and LNA #2.

To analyze the presence of polyA-tail of HSATII transcripts, total RNA was isolated and transcripts either were analyzed from total RNA fraction or underwent the polyA-tail enrichment using Oligotex mRNA Kit (Qiagen) according to the manufacturer's instructions. cDNA was made using either random hexamers or oligo-dT (Invitrogen by Thermo Fisher Scientific) together with Superscript™III Reverse Transcriptase Kit (Invitrogen by Thermo Fisher Scientific) according to the manufacturer's instructions. Quantitative PCR (qPCR) was performed using SYBR Green master mix (Applied Biosystems by Thermo Fisher Scientific) on the QuantStudio 6 Flex- Real Time PCR System (Applied Biosystems by Thermo Fisher Scientific).

Cell toxicity

HFFs were treated with LNA at concentrations ranging from 0 to 400 nM for 24h prior HCMV infection at a multiplicity of 1 $TCID_{50}$/cell or were mock infected. At indicated time points, the Cell Titer 96® AQueous One Solution Cell Proliferation Assay (Promega, Madison, WI) was performed according to the manufacturer's instructions. Absorbance was measured at 490 nm using the SpectraMax Plus 384 Microplate reader (Molecular Devices, Sunnyvale, CA). Data is presented as % viable cells and are presented as mean ± SD.

SUPPLEMENTARY FIGURES

Supplementary Fig. 1.

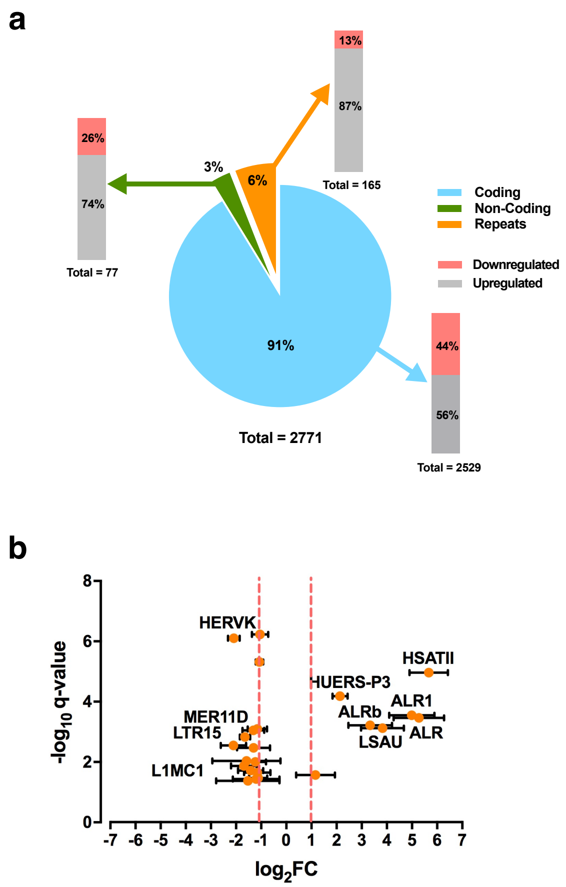

Supplementary Fig. 1. HCMV modulates non-coding RNAs, including transcripts of repeat elements. HFFs were infected with HCMV (AD169) at 3 $TCID_{50}$/cell and RNA samples were collected at 48 hpi. RNA was isolated and analyzed using RNA-seq. Differential expression of transcripts in infected cells was computed based on their expression in mock-infected cells. The q-value <0.05 and a fold change ±2 were used as significance thresholds**.** *n*=3. **a** The pie chart depicts differentially regulated coding, non-coding and repeat element transcripts in HCMV-infected fibroblasts at 48 hpi. The bar graphs represent a percent of upregulated (red bars) and downregulated (green bars) transcripts in each class. **b** Several classes of repeat elements are differentially regulated during HCMV infection. The graph presents cumulative RNA-seq data analysis from cells infected with AD169, TB40, FIX or TB40e strains of HCMV at 3 $TCID_{50}$/cell. Depicted are only repeat elements that are differentially expressed in each infection. The q-value was computed for an individual repeat element in the RNA-seq analysis of each infection experiment. The graph presents a log_2 fold change (FC) mean ± SD.

Supplementary Fig. 2.

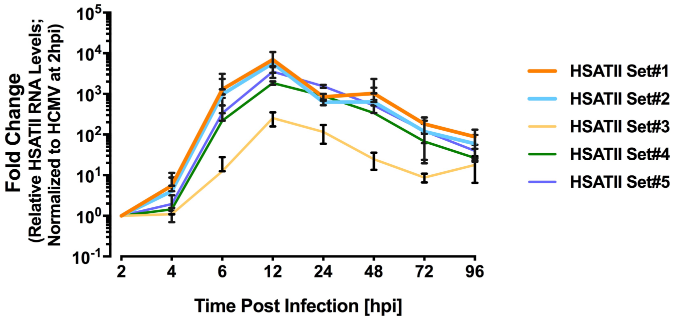

Supplementary Fig. 2. HCMV induces HSATII expression in epithelial cells. ARPE-19 cells were infected with HCMV (TB40-epi) at 3 $TCID_{50}$/cell and RNA samples were collected at the indicated time points. HSATII-specific primers were used in RT-qPCR analysis. GAPDH was used as an internal control. *n*=3. Data are presented as a fold change mean ± SD.

Supplementary Fig. 3.

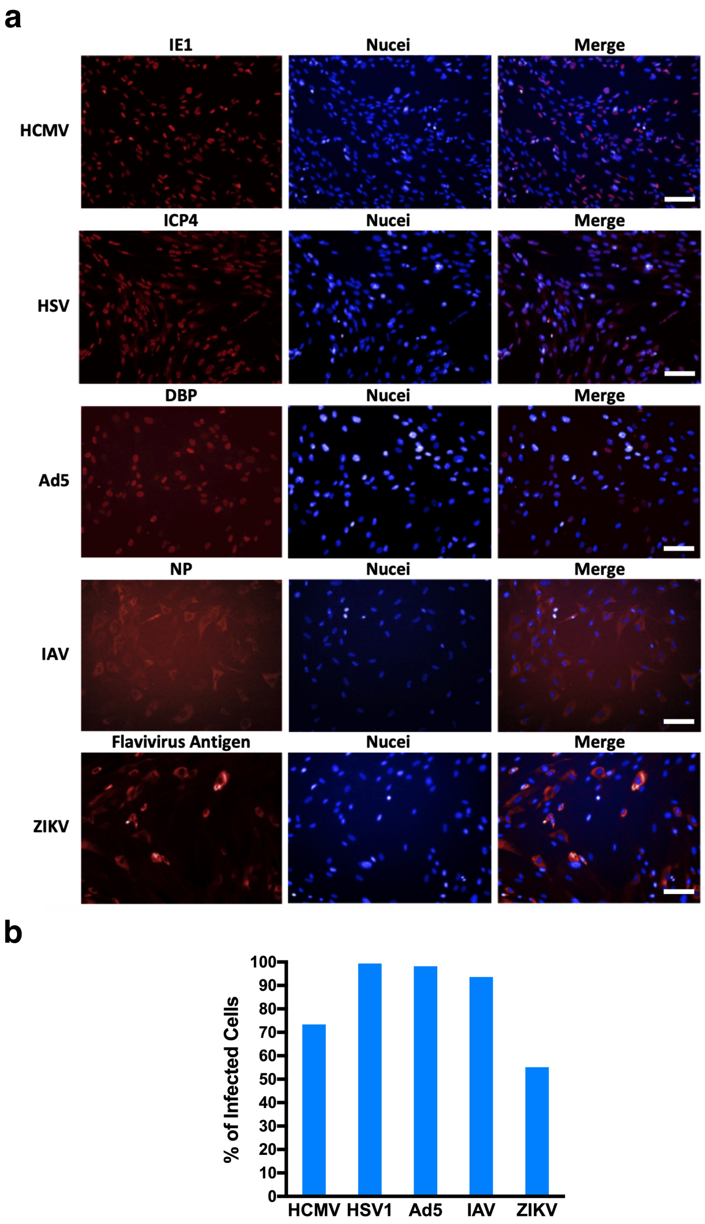

Supplementary Fig. 3. The efficiency of viral infections. HFFs were infected with HCMV (TB40/E-GFP; 3 $TCID_{50}$/cell), HSV1 (3 $TCID_{50}$/cell), Ad5 (10 FFU/cell), IAV (3 $TCID_{50}$/cell) or ZIKV (10 PFU/cell) and fixed at 24 hpi (HCMV and Ad5) or 12 hpi (HSV1 and IAV). Cells were stained for IE1 (HCMV), ICP4 (HSV1), DBP (Ad5), NP (IAV) or the flavivirus antigen (ZIKV) and nuclei were counterstained with the Hoechst stain. Cells were visualized (20X magnification) (**a**) and % of viral antigen-positive cells was calculated (**b**) using Operetta high-content imaging and analysis system. Scale bars: 100 µm.

Supplementary Fig. 4.

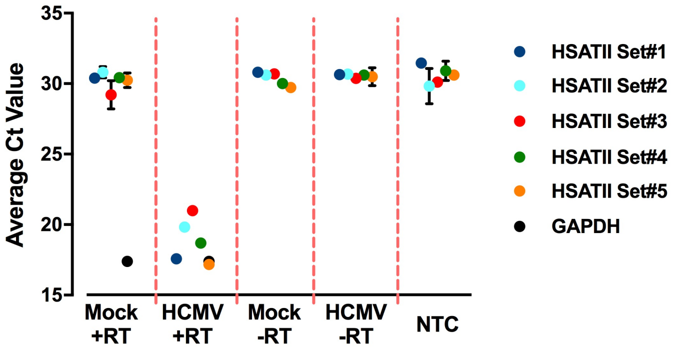

Supplementary Fig. 4. Lack of HSATII RNA:DNA hybrids in HCMV-infected cells. RNA samples were collected at 24 hpi from mock- or HCMV (TB40/E-GFP)-infected cells at 1 $TCID_{50}$/cell. RNA underwent RT reaction with or without reverse transcriptase and HSATII-specific primers were used to quantify HSATII expression by qPCR. GAPDH was used as an internal control. *n*=3. Data are presented as a mean ± SD.

Supplementary Fig. 5.

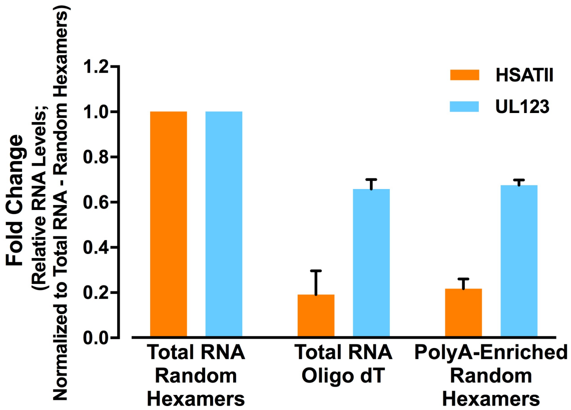

Supplementary Fig. 5. HSATII transcripts lack a polyA-tail. HFFs were infected with HCMV (TB40/E-GFP) at 1 $TCID_{50}$/cell and total RNA was collected at 24 hpi. Total RNA with or without enriching for polyA-tailed transcripts underwent RT reaction using random hexamers or oligo-dT. HSATII- and UL123-specific primers were used in RT-qPCR analysis. GAPDH was used as an internal control. *n*=3. Data are presented as a fold change mean ± SD.

Supplementary Fig. 6.

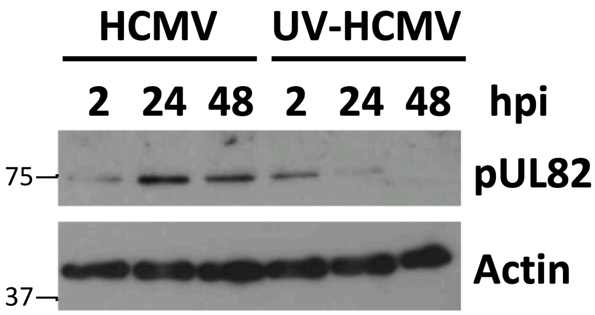

Supplementary Fig. 6. Analysis of UV-irradiated HCMV virions. HFFs were infected with untreated or UV-irradiated HCMV (TB40/E-GFP; 3 $TCID_{50}$/cell) and protein samples were collected at specified times. Protein levels were analyzed by western blotting using anti-pp71 antibody. Actin was used as a loading control.

Supplementary Fig. 7.

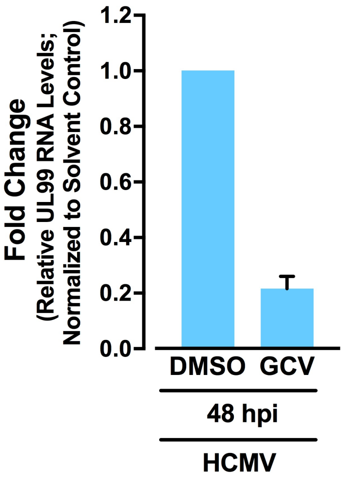

Supplementary Fig. 7. Effect of GCV on UL99 expression. HFFs were infected with HCMV (TB40/E-GFP; 1 $TCID_{50}$/cell) for 2h and then media was changed for one containing GCV or DMSO as a solvent control. RNA samples were collected at 48 hpi. RT-qPCR was performed using UL99-specific primers. GAPDH was used as an internal control. *n*=3. Data are presented as a fold change mean ± SD.

Supplementary Fig. 8.

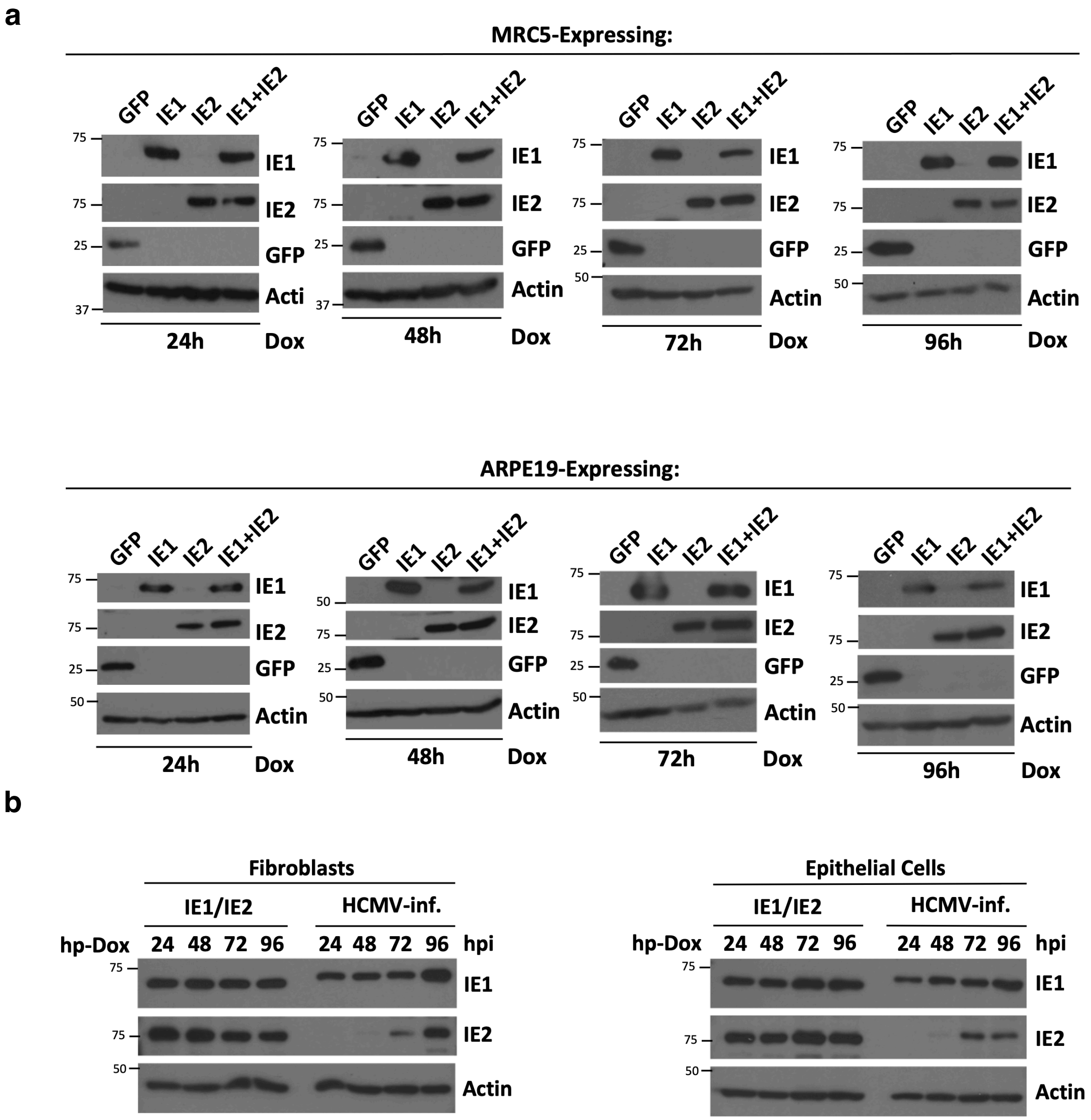

Supplementary Fig. 8. Analysis of GFP-, IE1-, IE2- or IE1/IE2-expressing cells. a Tetracycline-inducible MRC-5 and ARPE-19 cells were treated with doxycycline for 24, 48 or 72h or **b** HFF and ARPE19 cells were infected with HCMV (TB40/E-GFP and TB40-epi, respectively) at 1 $TCID_{50}$/cell. Protein samples were collected at indicated times. Protein levels were analyzed by western blotting using with anti-GFP, anti-IE1 or anti-IE2 antibodies. Actin was used as a loading control.

Supplementary Fig. 9.

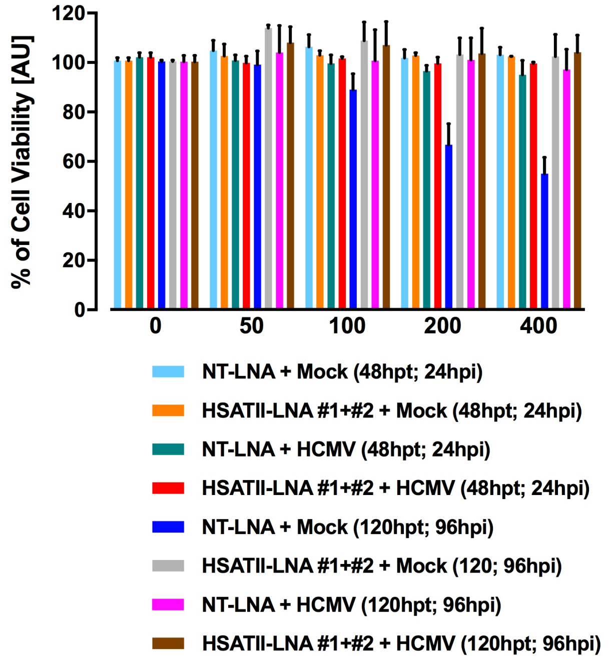

Supplementary Fig. 9. Effect of LNAs on cell viability. HFFs were transfected with increasing concentrations of NT-LNA or HSATII-LNAs. Cells were mock- or HCMV (TB40/E-GFP)-infected at 1 $TCID_{50}$/cell and cell viability was assessed at 48 h post LNA transfection (hpt) and 24 hpi or 120 hpt and 96 hpi. *n*=3. Data is presented as % viable cells mean ± SD.

Supplementary Fig. 10.

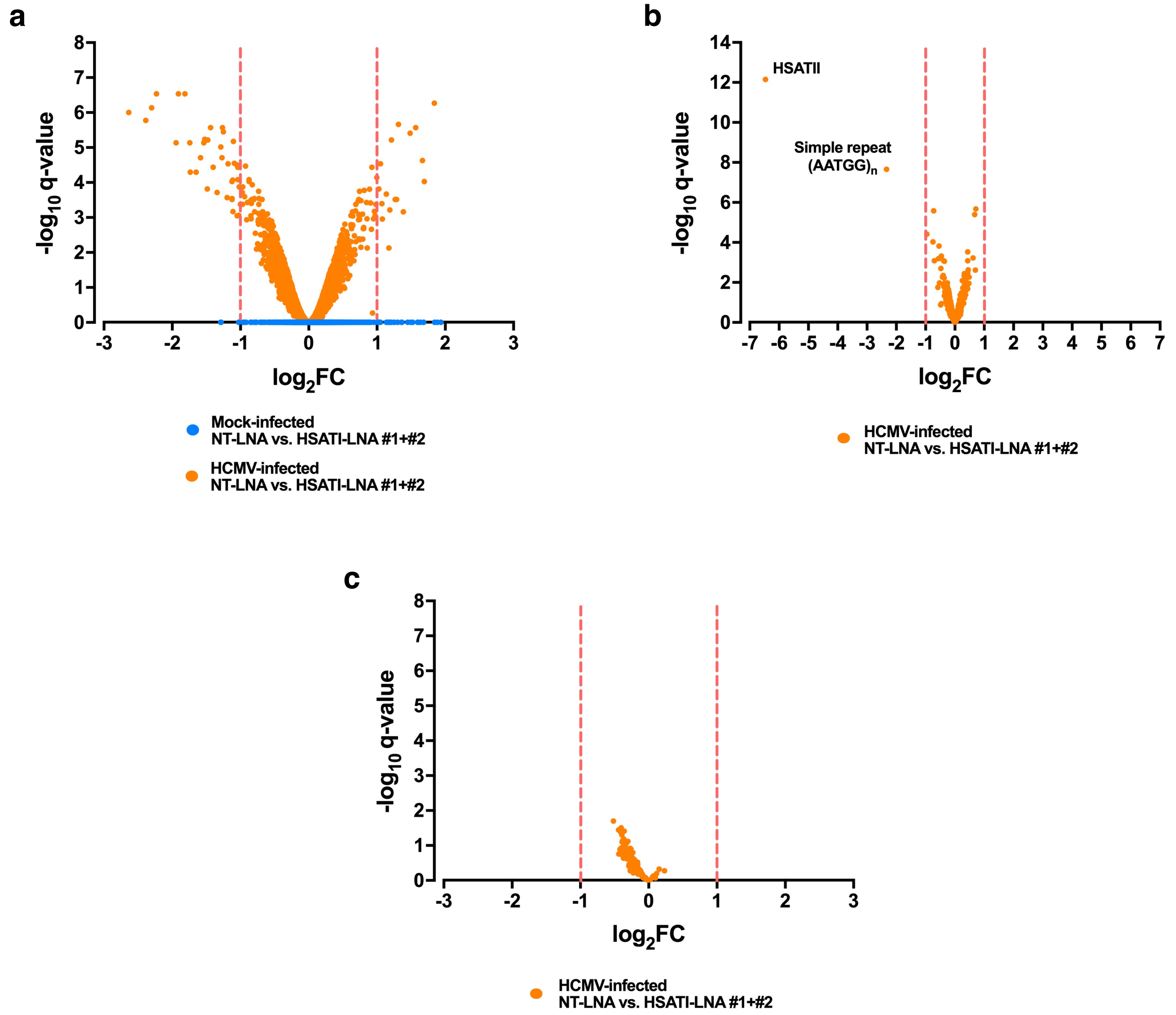

Supplementary Fig. 10. Effect of HSATII-specific LNAs on the expression of different types of transcripts. a HSATII-LNAs alter expression of protein-coding cellular RNAs in HCMV-infected fibroblasts. b HSATII-LNAs are highly specific for HSATII among non-coding cellular repeat RNAs. c HSATII-LNAs do not alter expression of HCMV transcripts at 24 hpi. (a,b,c) RNA samples were collected at 24 hpi from HFFs transfected with NT-LNA or HSATII-LNAs 24 h before mock or HCMV (TB40/E-GFP) infection at 1 $TCID_{50}$/cell. RNA was isolated and analyzed using RNA-seq. Differential expression of transcripts in HSATII-deficient cells was computed based on their expression in NT-LNA-transfected cells. Volcano plots were generated based on differential fold change expression of transcripts and the computed q-values between mock-infected, NT-LNA- and HSATII-LNA-transfected cells (blue dots) or between HCMV-infected, NT-LNA- and HSATII-LNA-transfected cells (orange dots). *n*=2. The graph presents a $\log_2$ fold change (FC).

Supplementary Fig. 11.

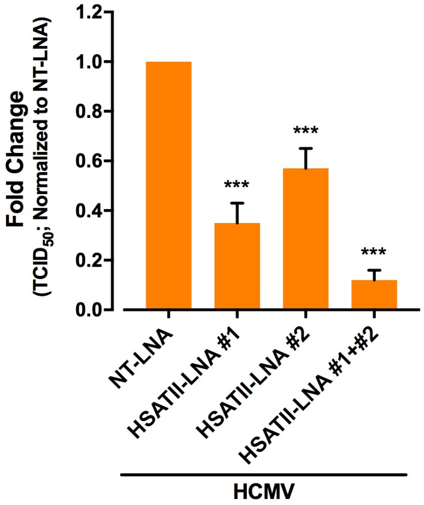

Supplementary Fig. 11. HSATII-specific LNAs affect HCMV yield. Media samples were collected at 96 hpi from HFFs transfected with NT-LNA, HSATII-LNA #1, HSATII-LNA #2 or the mixture of HSATII-LNAs 24 h before HCMV (TB40/E-GFP) infection at 1 $TCID_{50}$/cell. $TCID_{50}$/ml values were determined**.** *n*=3. Data are presented as a fold change mean ± SD. ****P*<0.001 by the unpaired, two-tailed *t*-test.

Supplementary Fig. 12.

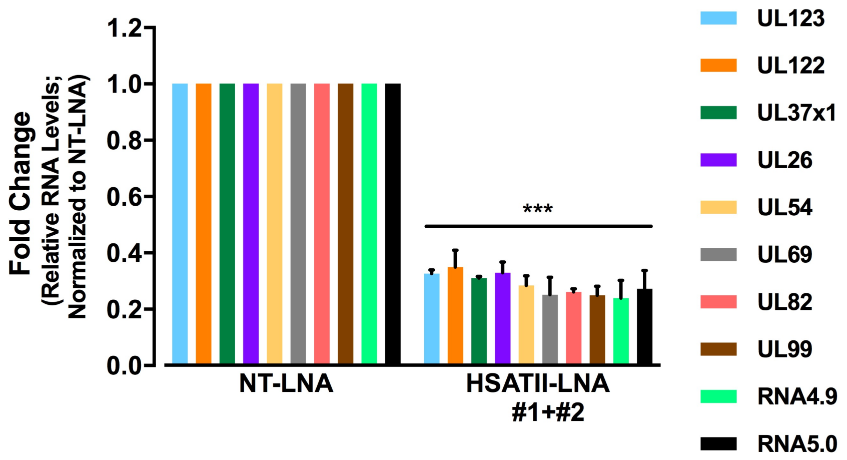

Supplementary Fig. 12. HSATII RNA is required for an efficient accumulation of all classes of viral transcripts. RNA samples were collected at 96 hpi from HFFs transfected with NT-LNA or HSATII-LNAs 24 h before HCMV (TB40/E-GFP) infection at 1 $TCID_{50}$/cell. RT-qPCR was performed using UL123, UL122, UL37x1, UL26, UL54, UL69, UL82, UL99, RNA4.9 and RNA5.0 specific primers. GAPDH was used as an internal control. *n*=3. Data are presented as a fold change mean ± SD. ****P*<0.001 by the unpaired, two-tailed *t*-test.

Supplementary Fig. 13.

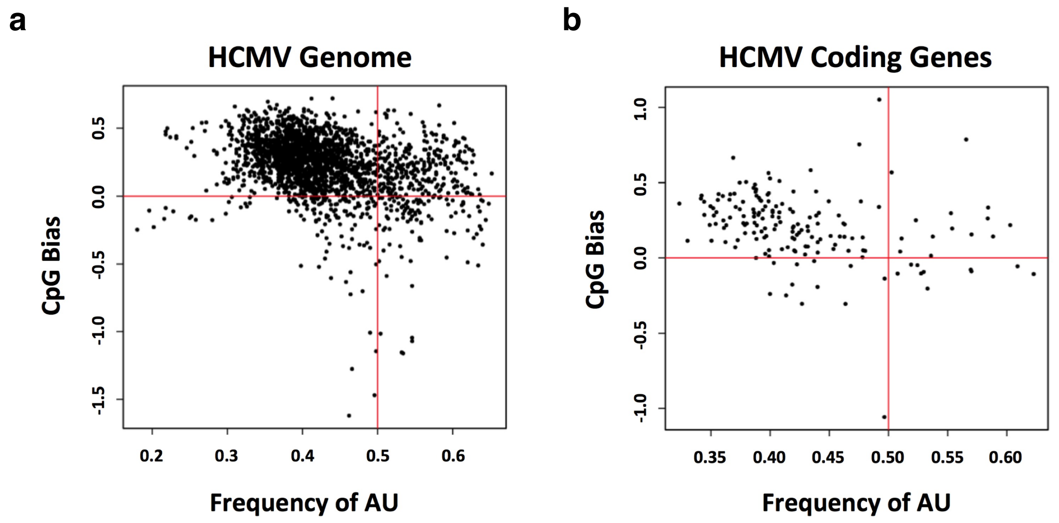

Supplementary Fig. 13. Comparison of HCMV genome and coding RNAs based on CpG bias and frequencies of AU. Histograms of forces (strength of statistical bias) on CpG for (a) HCMV genome and (b) HCMV coding RNAs compared to the frequency of AU-dinucleotides.

Supplementary Fig. 14.

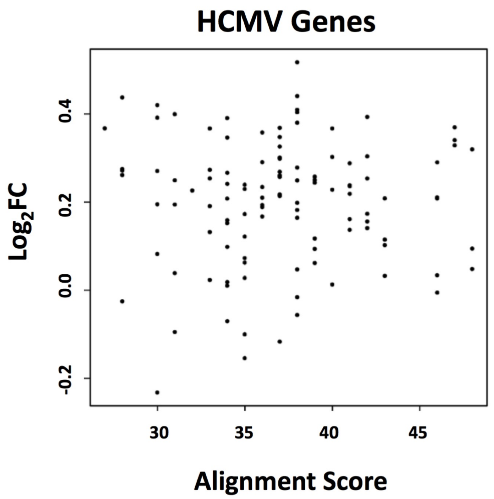

Supplementary Fig. 14. HSATII-LNAs do not affect differential expression of HCMV transcripts. Differential expression of HCMV transcripts at 24 hpi between NT-LNA- and HSATII-LNA-treated fibroblasts were plotted against the alignment score generated based on sequence similarity of the corresponding HCMV transcript sequence and HSATII-LNAs.

Supplementary Fig. 15.

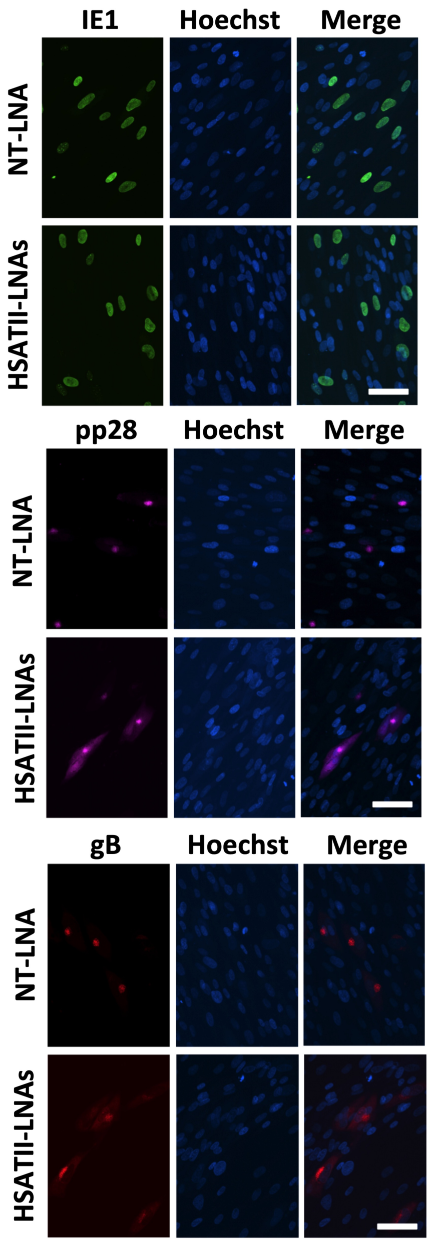

Supplementary Fig. 15. HSATII RNA affects cellular localization of HCMV late proteins. HFFs were transfected with NT-LNA or HSATII-LNAs 24 h before HCMV (TB40/E-GFP) infection at 1 $TCID_{50}$/cell. At 72 hpi, cells were fixed and stained for IE1, pp28 or gB and nuclei were counterstained with the Hoechst stain. Cells were visualized using Nikon Ti-E microscope with spinning disc (30X magnification). Scale bars: 100 µm.

Supplementary Fig. 16.

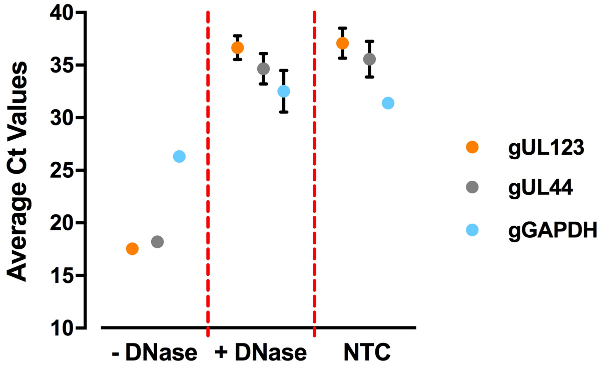

Supplementary Fig. 16. Effect of DNase on viral and cellular DNA levels. DNA samples were collected from HCMV (TB40/E-GFP)-infected HFFs at 1 $TCID_{50}$/cell and treated or not treated with DNase. qPCR was performed using gUL123, gUL44 and gGAPDH specific primers. *n*=3. Ct values are presented as a mean ± SD.

Supplementary Fig. 17.

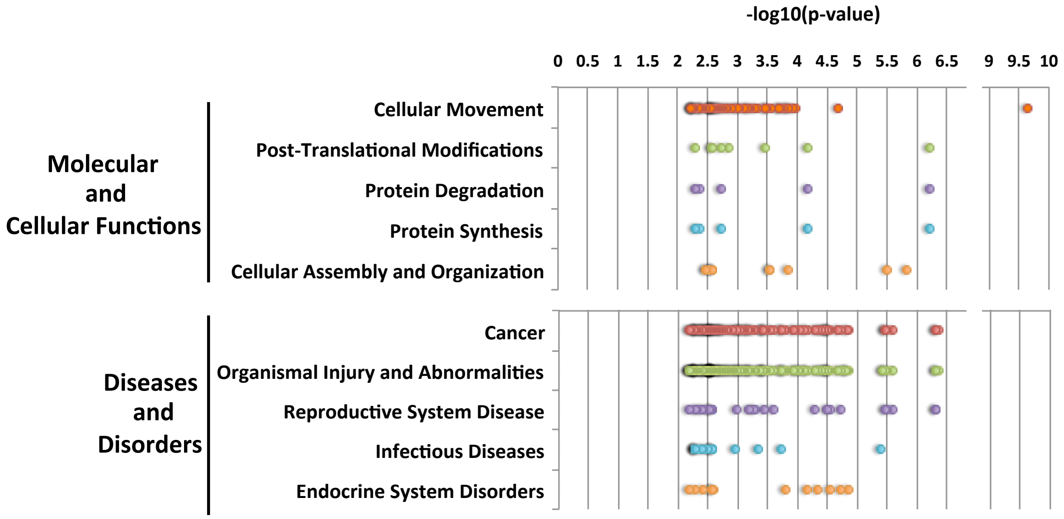

Supplementary Fig. 17. HSATII regulates several cellular processes. RNA was collected at 24 hpi from HFFs transfected with NT-LNA or HSATII-LNAs 24 h before mock or HCMV (TB40/E-GFP) infection at 1 $TCID_{50}$/cell. RNA was isolated and analyzed using RNA-seq. Differential expression of transcripts in HSATII-deficient cells was computed based on their expression in NT-LNA-transfected cells. A graphical representation of results of Core Analysis in IPA performed on a group of genes with expression significantly changed between HCMV-infected, NT-LNA- and HSATII-LNA-transfected HFFs. Genes were organized based on statistically enriched GO groups.

Supplementary Fig. 18.

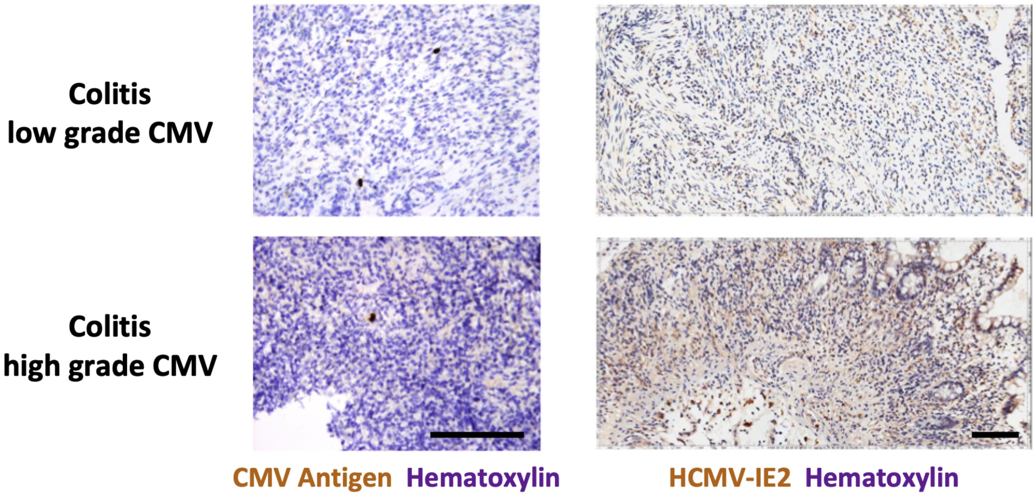

Supplementary Fig. 18. Representative colitis samples stained for a presence of CMV antigen. Paraffin-embedded sections of low and high grade CMV colitis commonly IHC stained for a mix of two CMV antigens (20X magnification) or IHC stained for HCMV IE2 (10X magnification). An intensely brown stain characterizes CMV-antigen positive cells. Nuclei were counterstained with hematoxylin (purple stain). Scale bars: 100 µm.

Supplementary Fig. 19.

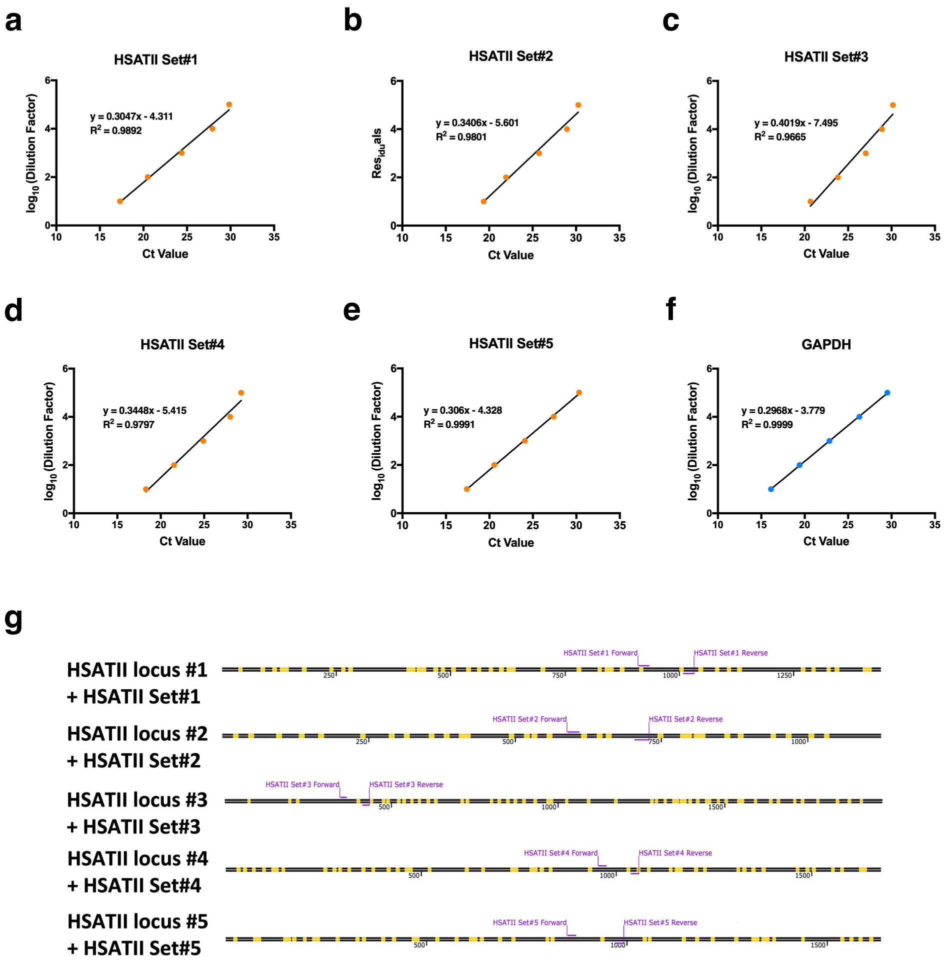

Supplementary Fig. 19. Development of RT-qPCR-based assay for a quantitative evaluation of HSATII expression. **a-e** – standard curves demonstrating a linear increase of HSATII amplicons with an increasing concentration of cDNA sample. **f** A standard curve demonstrating a linear increase of GAPDH amplicon with an increasing concentration of cDNA sample. **g** A graphical depiction of HSATII chromosomal loci showing binding locations of HSATII-specific primers. The orange color marks HSATII consensus sequence repeat.

Supplementary Fig. 20.

Uncropped – Fig. 3d

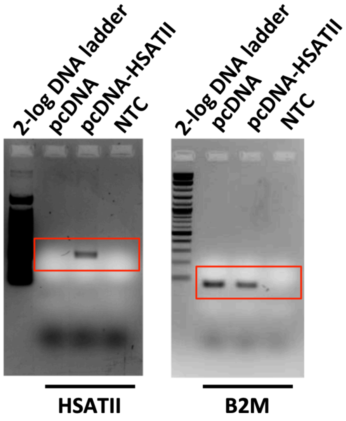

Uncropped – Fig. 4a

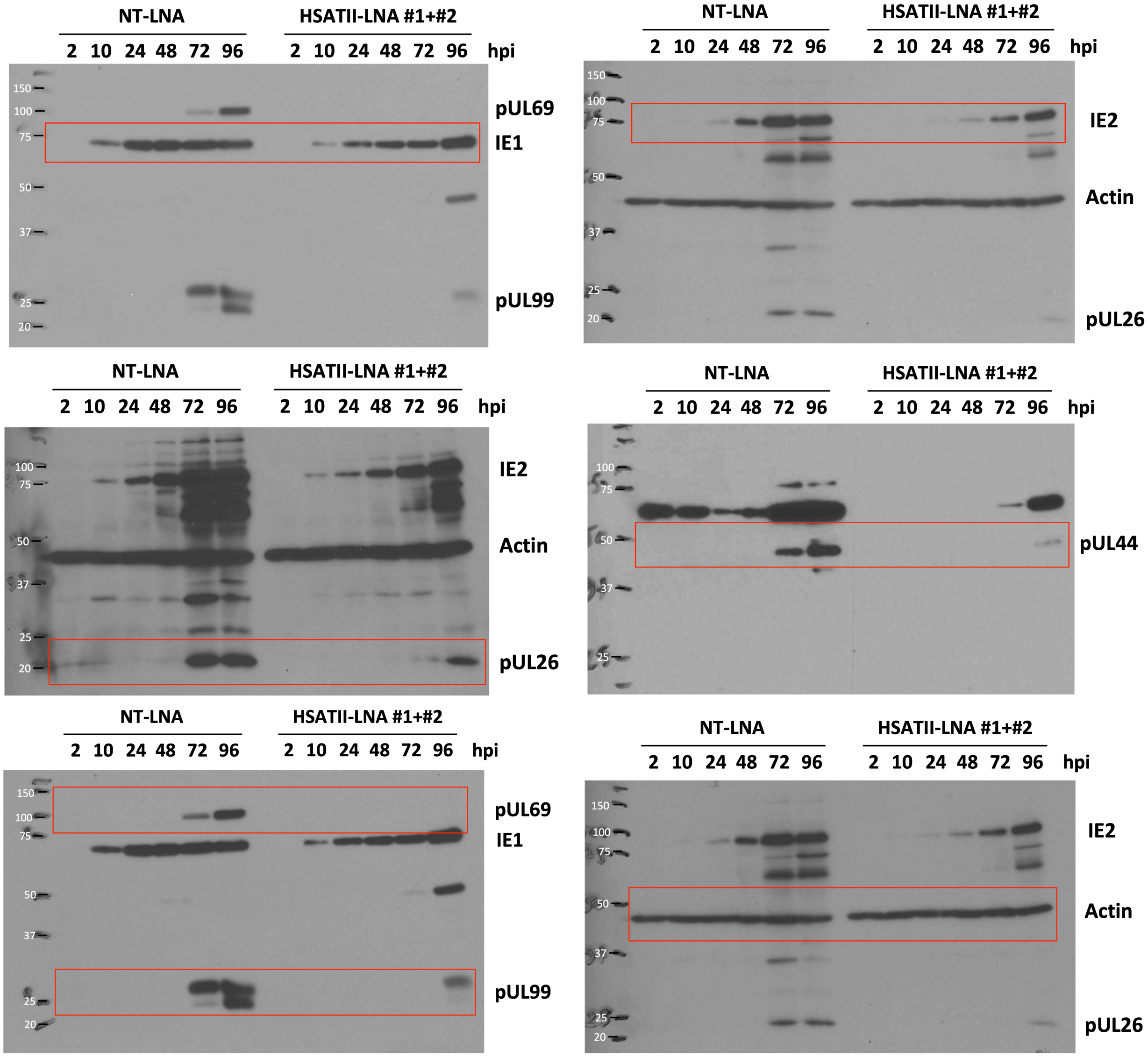

Supplementary Fig. 20. List of Uncropped images of western blots for Fig. 3d and 4a. The red boxes mark cropped areas.

SUPPLEMENTARY TABLES

Supplementary Table 1: Primer Sequences used in qPCR

Target Sequence	Forward Primer (5' to 3')	Reverse Primer (5'to 3')
HSV1 UL30[3]	CATCACCGACCCGGAGAGGGAC	GGGCCAGGCGCTTGTTGGTGTA
Ad5 E2A[4]	GTGTAGACACTTAAGCTCGCCTT	CTTCAAACTACTGCCTGACCAAGT
IAV Genome	CCACTGAAGTGGCATTTGGC	CTGTAGTGCTGGCTAAAACC
ZIKV Genome[5]	CCGCTGCCCAACACAAG	CCACTAACGTTCTTTTGCAGACAT
HCV Genome[6]	GTCTAGCCATGGCGTTAGTA	CTCCCGGGGCACTCGCAAGC
HSATII Set#1	CCAATGGAATCAGAAATAACCATCA	TCCTTTCATTTCCATTCAATGAGG
HSATII Set#2	TGTGATCATCATCGAACGGAC	ATGAGTCCTTCCTTTTCAATTTCAT
HSATII Set#3	TCGTGTCTATTCAAAGGTTCCA	ACGAGTGGAATCGATAGCCATAA
HSATII Set#4	GATTCCACTTGAGTCCGTTAG	GGAATCATCGTCGAATGGAG
HSATII Set#5	TTGGTGATTCCACTGGATTTCT	TCGGATGGAATCAATGAAGGGA
HCMV UL123	TGCTGTGCTGCTATGTCTTAGAGG	TTGGTTATCAGAGGCCGCTTGG
HCMV UL122	TGACCGAGGATTGCAACG	CGGCATGATTGACAGCCTG
HCMV UL37x1	TCCCGCCTTGGTTAAGA	ACTGGGCGTTGTTGAGCATA
HCMV UL26	CCAGCAGCTTCCAGTATTC	ACCTGGATCTGCCCTATC
HCMV UL54	TGCTTTCGTCGGTGCTCTCTAAG	TGTGCGGCAGGTTAGATTGACG
HCMV UL69	ACGAGTGTCAGAACGAGATGTGC	TGAAACGATAGGGTGCCAACGC
HCMV UL82	AGACGTCGAAGCGGTAACAACG	AGTCGTCAAGGCTCGCAAAGAC
HCMV UL99	ACGACAACATCCCTCCGACTTC	TCTGTTGCCGCTCCTCGTTATC
HCMV RNA4.9	TTGACAAGCGATGGAGGACC	TGAGCGGTTGTGTTGGATGA
HCMV RNA5.0	ACACCGTCAGGGAACACATC	GTGTATCGAGCCACCGTGAT
HSATII-pcDNA	CCGCCAGTGTGCTGGAATTC	GCCGCCAGTGTGATGGATATCA
GAPDH	CAAGAGCACAAGAAGAAGAGAG	CTACATGGCAACTGTGAGGAG
B2M	GCCCAAGATAGTTAAGTGGGATCG	TCCAAATGCGGCATCTTCAAACC

SUPPLEMENTARY REFERENCES

1 Greenbaum, B. D., Cocco, S., Levine, A. J. & Monasson, R. Quantitative theory of entropic forces acting on constrained nucleotide sequences applied to viruses. *Proc Natl Acad Sci U S A* **111**, 5054-5059 (2014).

2 Rice, P., Longden, I. & Bleasby, A. EMBOSS: the European Molecular Biology Open Software Suite. *Trends Genet* **16**, 276-277 (2000).

3 Kessler, H. H. *et al.* Detection of Herpes simplex virus DNA by real-time PCR. *J Clin Microbiol* **38**, 2638-2642 (2000).

4 Samad, M. A., Komatsu, T., Okuwaki, M. & Nagata, K. B23/nucleophosmin is involved in regulation of adenovirus chromatin structure at late infection stages, but not in virus replication and transcription. *J Gen Virol* **93**, 1328-1338 (2012).

5 Lanciotti, R. S. *et al.* Genetic and serologic properties of Zika virus associated with an epidemic, Yap State, Micronesia, 2007. *Emerg Infect Dis* **14**, 1232-1239 (2008).

6 Besnard, N. C. & Andre, P. M. Automated quantitative determination of hepatitis C virus viremia by reverse transcription-PCR. *J Clin Microbiol* **32**, 1887-1893 (1994).
